# Anisotropic Hyperelastic Modelling and Numerical Investigation of the Quasi-Static Compression Response of a Flexible Anti-Collision Airbag

**DOI:** 10.3390/polym18172166

**Published:** 2026-09-04

**Authors:** Yue Ni, Xiaobin Li, Zhenzhou Ye, Yiheng Zhang

**Affiliations:** 1 Ship Impact and Protection of Hubei Province Key Discipline Laboratory, Wuhan University of Technology, Wuhan 430063, China; 2 School of Naval Architecture, Ocean and Energy Power Engineering, Wuhan University of Technology, Wuhan 430070, China; 3 Department of Basic Courses, Naval University of Engineering, Wuhan 430033, China

**Keywords:** flexible anti-collision airbag, anisotropic hyperelasticity, parameter identification, localized quasi-static compression tests

## Abstract

Flexible anti-collision airbags are lightweight and highly deformable and are used for mitigating vessel–bridge collision loads, while reliable analysis requires accurate descriptions of the mechanical behaviour of the airbag materials. A three-layer single-chamber airbag comprising an inner thermoplastic polyurethane fabric (TPU) and two outer woven ultra-high-molecular-weight polyethylene (UHMWPE) reinforcing layers stitched locally by polyamide webbings is investigated. Uniaxial tensile tests are conducted on TPU and UHMWPE in the warp and weft directions, and anisotropic hyperelastic models are established. Based on model comparison and parameter identification, a three-parameter Yeoh base with a weak orthotropic correction is adopted for TPU, while a neo-Hookean base with a strong orthotropic correction is used for UHMWPE. Comparisons of the numerical and experimental results yield R^2^ values exceeding 0.9854 for force and 0.9995 for pressure, with relative differences of 4.183% and 0.348% at the maximum compression ratio η=0.7, respectively. The test results show that increasing the initial pressure from 0.12 to 0.14 MPa raises the force and pressure increments by 51.1% and 19.7%, respectively. Numerical analysis indicates that prescribed loading rates up to 10 m/min preserve the quasi-static response at η of 0.7 with less than approximately 1% deviation. At η=0.7, increasing the axial loading length ratio $\eta_{L}$ from 0.2 to 0.6 raises the force by 208.7%, while the unit-length force changes by only 7.56%, whereas a larger airbag aspect ratio Λ increases the force but reduces the unit-length force and pressure increment under constant $\eta_{L}$. When the axial eccentricity reaches 0.20, the force and pressure increments rise by 45.5% and 31.3%, respectively. The established models provide a reliable basis for the localized compression analysis of the anisotropic multilayer airbag.

## 1. Introduction

Inflatable airbags serve as passive protective devices in automotive occupant-restraint systems, spacecraft landing, and emergency rescue operations [1,2,3]. Airbags have also been employed for ship launching and investigated as pneumatic fenders to safeguard against ship collisions in marine engineering [4,5]. Flexible airbags combine lightweight construction and compact stowage with a substantial deformation stroke, and the mechanical response varies with the inflation conditions and geometric configuration [6,7].

The cushioning response of airbags is commonly investigated using analytical models, numerical simulations and experiments. Existing analytical models typically derive the relationship between force and deformation based on idealized airbag geometry, and subsequent studies have further considered the effects of airbag shape and venting conditions [8]. Finite element models have also been used to reproduce large deformations, internal pressure changes, and contact during compression or impact [9,10]. In addition, quasi-static tests provide experimental data for evaluating numerical models of inflatable membrane structures and reinforced ship launching airbags [11,12]. These studies clarify the effects of geometry, inflation conditions and loading configurations on force and pressure responses. However, accurate analysis of multilayer flexible airbags also requires appropriate descriptions of the nonlinear and directionally dependent response of the airbag materials.

Thermoplastic polyurethane (TPU) exhibits pronounced nonlinear tensile behaviour at large deformation, and the response is commonly described by hyperelastic strain energy functions calibrated based on experimental curves [13,14]. Ultra-high-molecular-weight polyethylene (UHMWPE) combines low density with high tensile performance and is widely used as a lightweight reinforcement material [15]. Directional tensile tests on airbag fabrics further show nonlinear and direction-dependent responses in the warp and weft directions [16,17]. Therefore, anisotropic hyperelastic models based on tensor invariants are proposed to describe the nonlinear behaviour and deformation responses along principal material directions [18]. Guo et al. [19] decompose the strain energy into contributions from yarn extension and interaction, and identify the model parameters with uniaxial tensile and shear tests. Yao et al. [20] subsequently develop an anisotropic hyperelastic model considering the interaction between the two yarn directions under biaxial tension. Invariant-based models for thermoplastic woven prepregs and two-dimensional fabrics are further established and evaluated through numerical simulations by Gong et al. [21] and Song et al. [22], respectively. These studies provide a basis for modelling fabric materials, but the form and parameters of directional terms remain dependent on measured material response. Accordingly, a reliable analysis of multilayer airbags containing TPU and UHMWPE requires separate material descriptions to reflect the nonlinear and directional behaviours.

**Figure 1:**
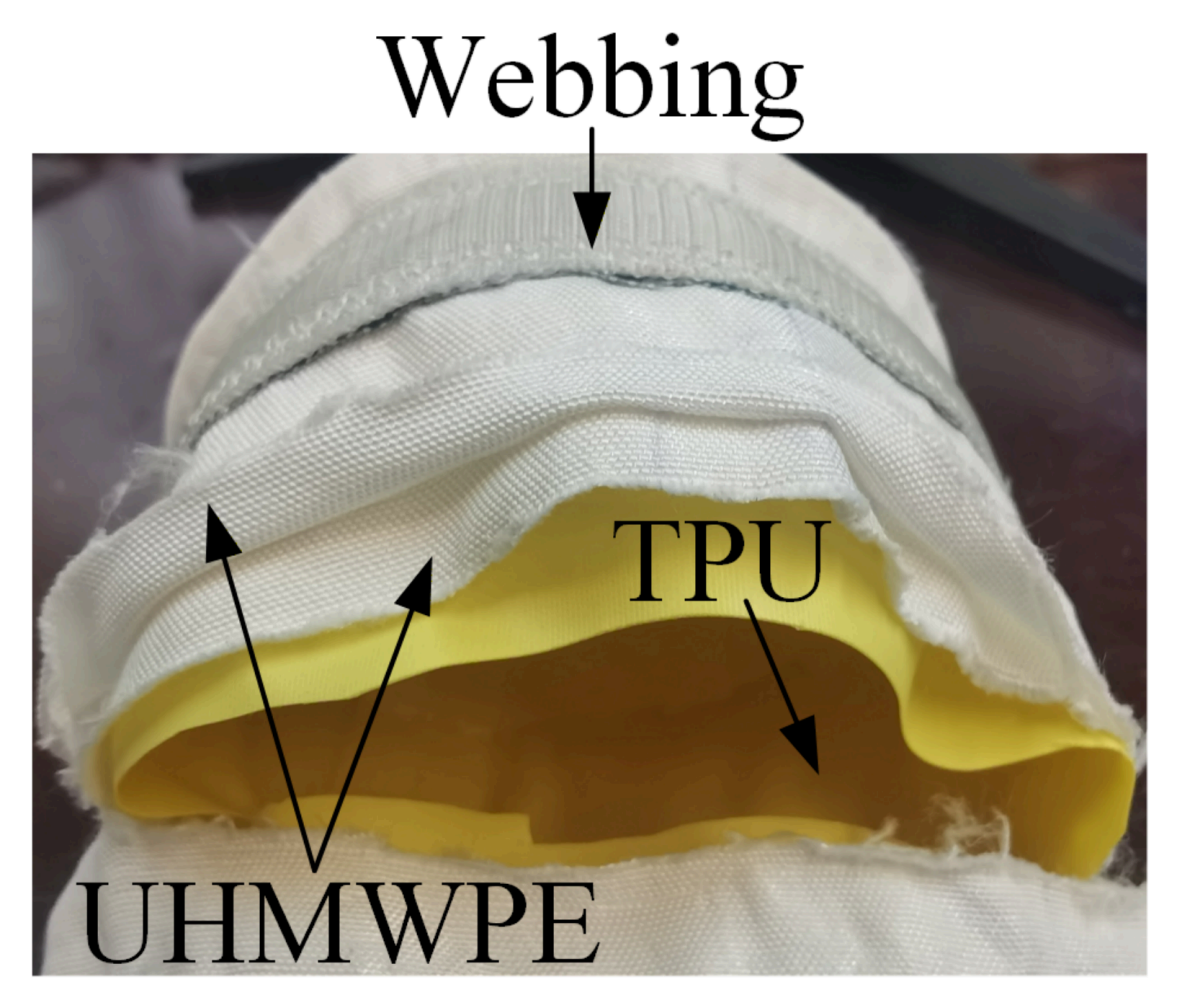
Material arrangement of the airbag.

Accordingly, this study develops orthotropic hyperelastic material models for a multilayer flexible anti-collision airbag to accurately predict the cushioning response under localized quasi-static compression. The airbag examined in this study is a three-layer airbag consisting of an inner continuous TPU fabric and two outer woven UHMWPE fabric layers. The layers are not directly bonded but connected by seven equally spaced stitched polyamide webbings. The material arrangement of the airbag is shown in Figure 1. Uniaxial tensile tests are first performed on the TPU and UHMWPE layers in the warp and weft directions, and combinations of different isotropic bases and direction-dependent orthotropic corrections are identified and compared. Localized compression tests are conducted at various initial pressures and loading rates and are then used to validate the numerical model and material parameters. Finally, the validated model is employed to investigate the effects of loading rate, axial loading length ratio, airbag aspect ratio, and axial eccentricity on the compression response.

## 2. Uniaxial Tensile Behaviour and Anisotropic Hyperelastic Modelling

Uniaxial tensile tests are performed on the TPU and UHMWPE layers in the warp and weft directions of the tested airbag. The anisotropic hyperelastic models for both materials are established based on their nonlinearity and directional dependence. Within the framework of incompressible hyperelasticity, isotropic bases and orthotropic correction terms are selected by simultaneous parameter identification. The selected models are implemented and validated, and the identified parameters are used to perform a finite element analysis of the flexible anti-collision airbag.

### 2.1. Uniaxial Tensile Tests

**Figure 2:**
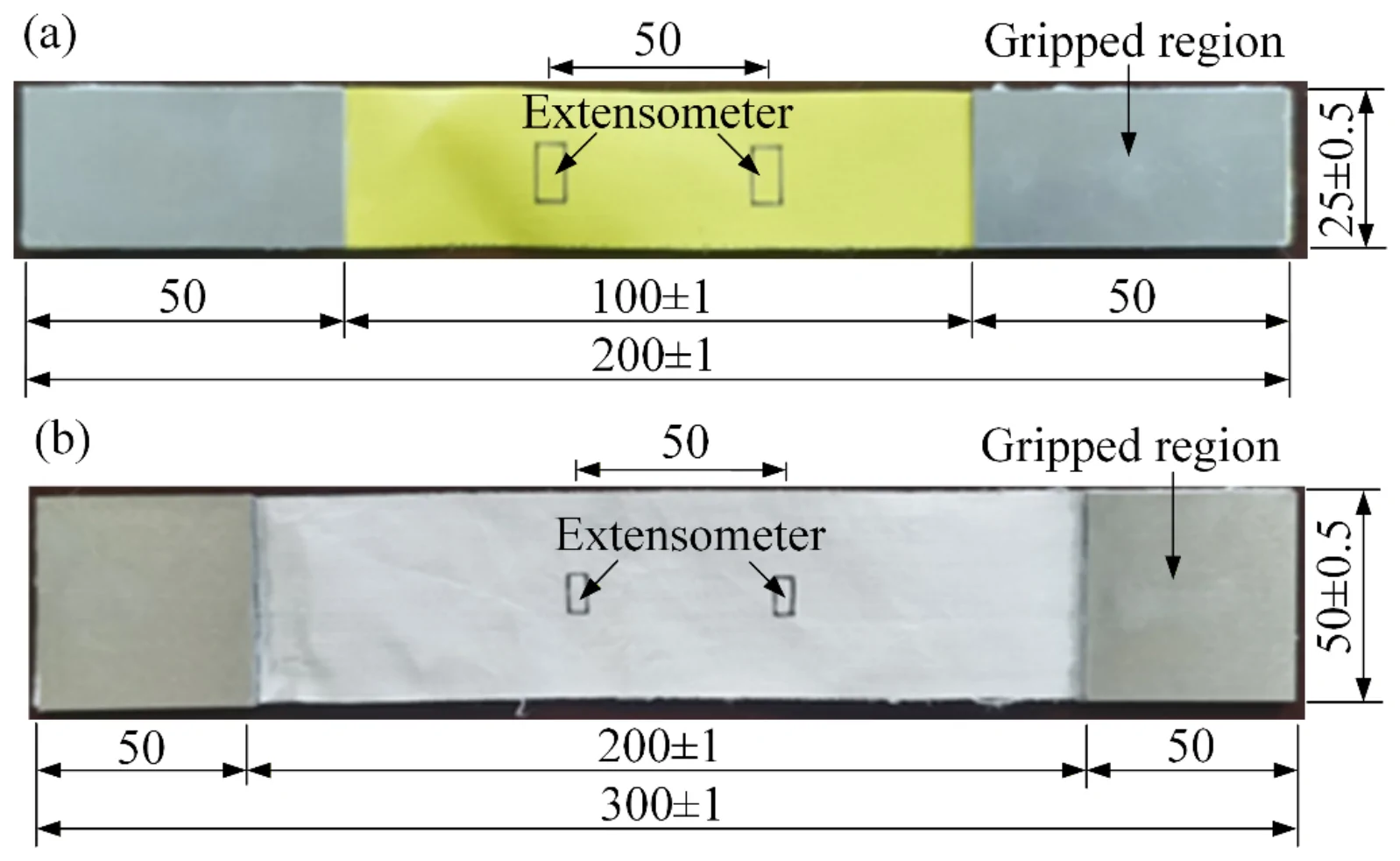
Dimensions of the uniaxial tensile specimens: (**a**) TPU; (**b**) UHMWPE (mm).

The test airbag has a three-layer structure with an inner TPU and two outer UHMWPE woven fabric layers. The TPU layer forms a continuous airtight boundary and accommodates the large deformation associated with inflation and deflation [13,23], while the UHMWPE fabrics reinforce the airbag structure due to their low density, high specific strength and impact resistance [24,25]. The TPU and UHMWPE strip specimens are supplied by the airbag manufacturer (Hubei, China), according to the dimensions shown in Figure 2. The TPU specimens have an effective width of 25 ± 0.5 mm, a total length of 200 ± 1 mm, and an initial free length of 100 ± 1 mm [26,27]. Corresponding dimensions of the UHMWPE specimens are 50 ± 0.5 mm, 300 ± 1 mm, and 200 ± 1 mm [28,29], respectively. Separate specimens are prepared in the warp (material direction 1) and weft (material direction 2), with the specimen longitudinal axis parallel to the corresponding material direction, and five specimens are tested for each material and loading direction. The measured average thicknesses are 0.30 mm for TPU and 0.38 mm for UHMWPE. Aluminum tabs are attached to both ends of the specimens and cured under clamping pressure for 24 h to limit local compression and slippage in the grips.

**Figure 3:**
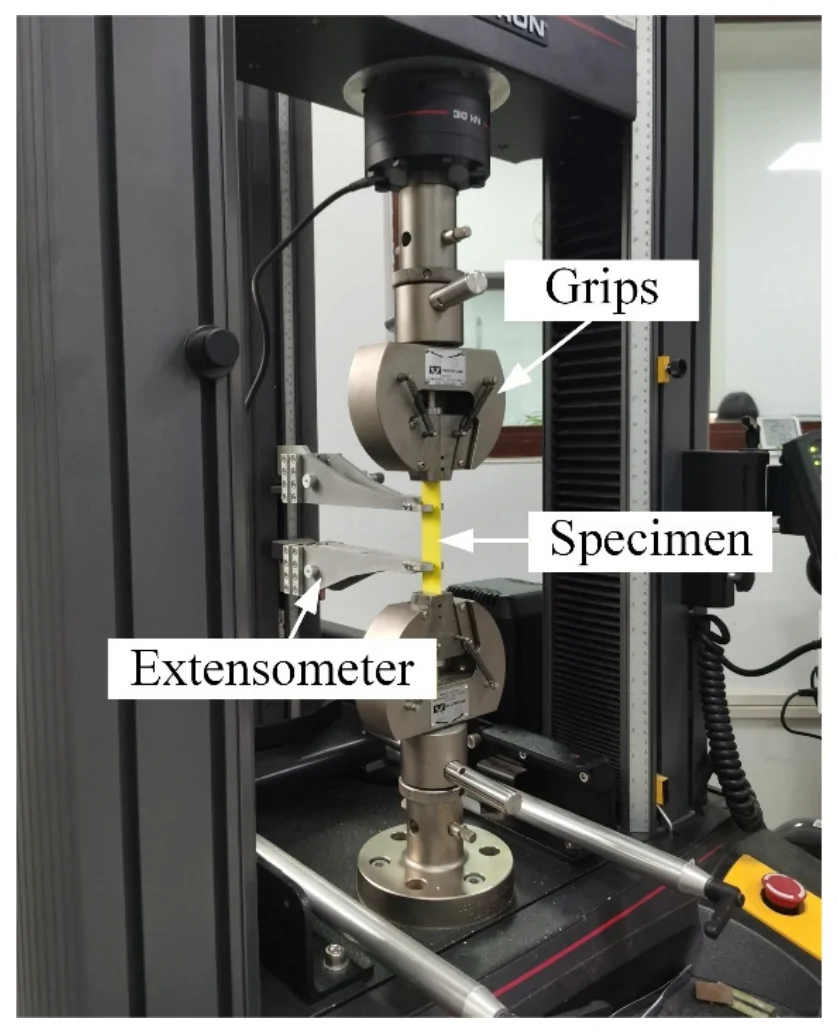
Experimental setup for the uniaxial tensile tests.

The tests are performed using an Instron 5967 universal testing machine, as shown in Figure 3. Each specimen is aligned with the loading axis, with the lower grip fixed and the upper grip moving at a constant speed of 50 mm/min for TPU and 100 mm/min for UHMWPE. The axial force and extension of the gauge length are recorded simultaneously.

The tensile responses are characterized by the nominal stress and stretch ratio

(1) $$P_{\exp}=\frac{F_{t}}{A_{0}},\;\;\lambda =\frac{L}{L_{0}}.$$

where $F_{t}$ is the measured axial force, $A_{0}=b_{0}t_{0}$ is the initial cross-sectional area, $b_{0}$ and $t_{0}$ are the initial width and average thickness, $L_{0}$ and L are the initial and current lengths of the measurement region, respectively. The five replicate curves are averaged over their common continuous loading interval, and the resulting average curve is used for constitutive identification.

**Figure 4:**
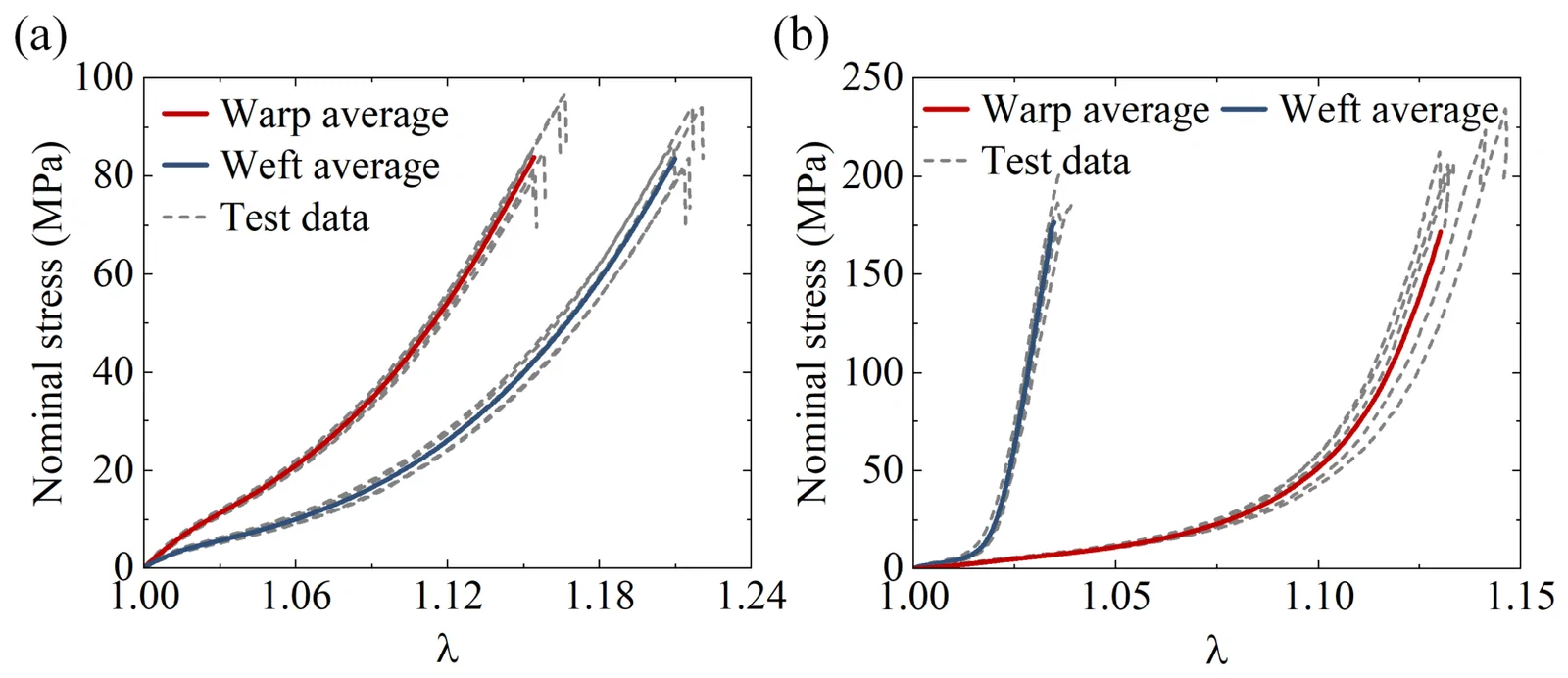
Uniaxial tensile responses in the warp and weft directions: (**a**) TPU; (**b**) UHMWPE. Grey dashed lines represent the test data, while red and blue solid lines represent the warp and weft averages, respectively.

The TPU curves show that stiffness gradually increases during stretching in both directions (Figure 4a), and the five repeats show similar trends, indicating good reproducibility. The peak nominal stress and corresponding strain are 88.20 ± 7.14 MPa and 15.93 ± 0.57%, respectively, for the warp direction, while those for the weft direction are 87.83 ± 6.02 MPa and 21.51 ± 0.40%. The difference between the peak nominal stresses is only 0.4%, whereas the peak strain in the weft direction is about 35% higher.

As shown in Figure 4b, UHMWPE exhibits a much stronger directional dependence. The warp response contains an extended region of low slope followed by a rapid increase, whereas the weft response reaches high stresses within a smaller deformation range. The strain at peak stress is about 3.8 times that in the weft direction, and the curvature evolution in the two directions also differs significantly. The repeated curves remain concentrated during the initial loading stage and show some dispersion at larger deformation while maintaining the same trends.

Moreover, the abrupt stress drop at the ends of the curves corresponds to macroscopic fracture. Since no material failure is observed in subsequent quasi-static compression tests, parameter identification is limited to the stable loading range before fracture. Damage evolution is not considered in the present model.

### 2.2. Anisotropic Hyperelastic Models

The uniaxial tensile results show pronounced nonlinear responses for both TPU and UHMWPE, with different degrees of directional dependence. The curve shapes and peak stresses are similar in the warp and weft directions for TPU, whereas UHMWPE exhibits much bigger directional differences in the deformation range and stress evolution. Accordingly, an orthotropic hyperelastic model based on invariants is adopted [30,31], in which the total strain energy is decomposed into an isotropic contribution and an orthotropic correction.

(2) $$W=W_{\mathrm{iso}}+W_{\mathrm{ortho}}.$$

The isotropic contribution represents the basic nonlinear response common to both material directions, while orthotropic corrections account for the measured difference between the warp and weft responses. The TPU curves show similar shapes and peak stresses, so the weak orthotropic form is introduced as a limited directional modification to the common isotropic response. For UHMWPE, the deformation ranges and curvature evolutions differ substantially, and the strong orthotropic form therefore assigns independently adjustable direction-dependent functions. The corresponding stresses are

(3) $$S=2\frac{\partial W}{\partial C},\;\;P=FS,\;\;\sigma =\frac{1}{J}FSF^{T}.$$

where F denote the deformation gradient, $C=F^{T}F$ is the right Cauchy–Green tensor, and J=detF is the volume ratio. Under the incompressibility constraint, J=1 [32], the local material directions $e_{1}$ and $e_{2}$ correspond to the warp and weft directions, respectively. The associated invariants are defined as

(4) $$\begin{array}{l} I_{1}=\mathrm{tr}C,\;\;I_{2}=\frac{1}{2}\left[{\left(\mathrm{tr}C\right)}^{2}-\mathrm{tr}(C^{2})\right],\\ I_{4}^{\left(ii\right)}=\mathrm{tr}(CG_{i}),\;\;I_{5}^{\left(ii\right)}=\mathrm{tr}(C^{2}G_{i}). \end{array}$$

where $G_{i}=e_{i}⊗e_{i},i=1,2$.

To describe the difference between the warp and weft responses of TPU, the weak orthotropic correction is formulated in terms of the Green–Lagrange strain tensor E=(C−I)/2 and the directional-difference tensor $M=G_{1}-G_{2}$.

According to the irreducible representation theory for two symmetric second-order tensors [33,34], and retaining terms up to second order in E, the weak orthotropic strain-energy correction $W_{\mathrm{ani}}^{w}(E,M)$ can be constructed from trEtr(M⋅E), $\mathrm{tr}E\mathrm{tr}(M^{2}\cdot E)$, $\mathrm{tr}(M\cdot E)\mathrm{tr}(M^{2}\cdot E)$, $\mathrm{tr}(M\cdot E^{2})$, $\mathrm{tr}(M^{2}\cdot E^{2})$, ${\left[\mathrm{tr}(M\cdot E)\right]}^{2}$, and ${\left[\mathrm{tr}(M^{2}\cdot E)\right]}^{2}$.

To determine the coefficients of the expansion, the orthotropic stiffness at the reference configuration is decomposed into an isotropic reference part $C_{\mathrm{iso}}^{V}$, and a residual directional correction $\Delta C^{V}$. The decomposition is written as

(5) $$C_{\mathrm{ortho}}^{V}=C_{\mathrm{iso}}^{V}+\Delta C^{V},\;\;\Delta C^{V}=\left[\begin{array}{cccccc} 0 & k_{12} & k_{13} & 0 & 0 & 0\\ k_{12} & k_{22} & k_{23} & 0 & 0 & 0\\ k_{13} & k_{23} & k_{33} & 0 & 0 & 0\\ 0 & 0 & 0 & 0 & 0 & 0\\ 0 & 0 & 0 & 0 & k_{55} & 0\\ 0 & 0 & 0 & 0 & 0 & k_{66} \end{array}\right].$$

where $k_{ij}$ are the orthotropic correction parameters to be identified.

Accordingly, the weak orthotropic strain-energy correction adopted for TPU is obtained as

(6) $$\begin{array}{c} W_{\mathrm{ani}}^{w}=a_{0}I_{1}+a_{1}I_{1}I_{4}^{\left(11\right)}+a_{2}I_{1}I_{4}^{\left(22\right)}+\frac{b_{11}}{2}\;{\left(I_{4}^{\left(11\right)}\right)}^{2}+\frac{b_{22}}{2}\;{\left(I_{4}^{\left(22\right)}\right)}^{2}\\ \;\;\;+b_{12}I_{4}^{\left(11\right)}I_{4}^{\left(22\right)}+d_{1}I_{4}^{\left(11\right)}+d_{2}I_{4}^{\left(22\right)}-\frac{k_{55}}{2}I_{5}^{\left(11\right)}-\frac{k_{66}}{2}I_{5}^{\left(22\right)}+c_{0}. \end{array}$$

where

$$\begin{array}{l} a_{0}=\frac{k_{33}}{2}-\frac{k_{13}+k_{23}}{4}-k_{55}-k_{66},\;a_{1}=\frac{k_{55}+k_{66}}{2}+\frac{k_{13}-k_{33}}{4},\;a_{2}=\frac{k_{55}+k_{66}}{2}+\frac{k_{23}-k_{33}}{4},\\ b_{11}=\frac{k_{33}}{4}-\frac{k_{13}}{2}-k_{66},\;b_{12}=\frac{k_{12}-k_{13}-k_{23}+k_{33}}{4}-\frac{k_{55}+k_{66}}{2},\\ b_{22}=\frac{k_{22}+k_{33}}{4}-\frac{k_{23}}{2}-k_{55},\;d_{1}=\frac{k_{23}-k_{12}+k_{33}}{4},\;d_{2}=\frac{k_{13}-k_{12}-k_{22}+k_{33}}{4},\\ c_{0}=\frac{3}{2}(k_{55}+k_{66})-k_{33}+\frac{k_{12}+k_{13}+k_{23}}{4}+\frac{k_{22}}{8}. \end{array}$$

In contrast, the warp and weft responses of UHMWPE differ not only in peak stress but also in the low-stiffness region, effective deformation range, and curvature evolution. Therefore, a strong orthotropic correction is adopted for UHMWPE, with independently adjustable functions assigned to the two material directions. Based on the Cayley–Hamilton relation, the mixed invariant $K_{5i}$ [33] is introduced, and the general form of the strong orthotropic strain-energy correction is [35]

(7) $$W_{\mathrm{ani}}^{s}=\sum_{i=1}^{2}D_{i}\left[W_{i}^{I}\;\left(I_{4}^{\left(ii\right)}\right)+W_{i}^{K}\;\left(K_{5i}\right)\right].$$

where $K_{5i}=I_{5}^{\left(ii\right)}-I_{1}I_{4}^{\left(ii\right)}+I_{2}$. The functions $W_{i}^{I}$ and $W_{i}^{K}$ can be defined independently for the warp and weft directions. To characterize the distinct rapid-stiffening responses of UHMWPE in the two directions, exponential forms are adopted [36]

(8) $$W_{i}^{I}\;\left(I_{4}^{\left(ii\right)}\right)=\frac{e^{A_{i}(I_{4}^{\left(ii\right)}-1)}-1}{A_{i}},W_{i}^{K}\;\left(K_{5i}\right)=\frac{e^{B_{i}(K_{5i}-1)}-1}{B_{i}}.$$

The strong orthotropic strain-energy correction for UHMWPE is therefore expressed as

(9) $$W_{\mathrm{ani}}^{s}=\sum_{i=1}^{2}D_{i}\left[\frac{e^{A_{i}(I_{4}^{\left(ii\right)}-1)}-1}{A_{i}}+\frac{e^{B_{i}(K_{5i}-1)}-1}{B_{i}}\right].$$

where $A_{i}$, $B_{i}$, and $D_{i}$ are the orthotropic correction parameters to be identified.

Accordingly, the total strain-energy functions for the two materials are therefore written as

(10) $$W_{\mathrm{TPU}}=W_{\mathrm{iso}}+W_{\mathrm{ani}}^{w},\;\;W_{\mathrm{UHMWPE}}=W_{\mathrm{iso}}+W_{\mathrm{ani}}^{s}.$$

### 2.3. Parameter Identification and Model Selection

The isotropic contribution characterizes the basic nonlinear response common to both material directions. Based on established weak and strong orthotropic corrections, six classical isotropic hyperelastic models are considered, including the neo-Hookean (NH) model [37], the Gent model [38], the two-parameter, five-parameter, and nine-parameter Mooney–Rivlin models, denoted as MR2, MR5, and MR9, respectively [39,40], and the three-parameter Yeoh model (Y3) [41]. The corresponding strain-energy functions are given by

(11) $$\begin{array}{l} W_{\mathrm{iso}-\mathrm{NH}}=C_{10}(I_{1}-3),\\ W_{\mathrm{iso}-G}=-\frac{C_{10}}{2}G_{1}\ln\;\left(1-\frac{I_{1}-3}{G_{1}}\right),\\ W_{\mathrm{iso}-\mathrm{MR}N}=\sum_{1\leq i+j\leq N}C_{ij}{\left(I_{1}-3\right)}^{i}{\left(I_{2}-3\right)}^{j},\;\;N=1,2,3,\\ W_{\mathrm{iso}-Y3}=\sum_{i=1}^{3}C_{i0}{\left(I_{1}-3\right)}^{i}. \end{array}$$

where $C_{ij}$, and $G_{1}$ are the isotropic parameters to be identified. The Mooney–Rivlin polynomial orders N=1, 2, and 3 correspond to MR2, MR5, and MR9, respectively. Each isotropic model is combined with the weak orthotropic correction for TPU and strong orthotropic correction for UHMWPE. The parameters of the resulting models are identified jointly from both warp and weft test curves.

Under the incompressibility constraint, the augmented strain-energy function is expressed as

(12) Ψ=W−p(J−1).

where p is the Lagrange multiplier. For uniaxial loading along a material principal direction i, the axial nominal stress is given by

(13) $$P_{ii}=\frac{\partial W}{\partial \lambda_{i}}-p\lambda_{j}\lambda_{k}.$$

For each prescribed axial stretch $\lambda_{i}$, the two lateral stretches and p are obtained by solving the incompressibility condition together with the two traction-free lateral conditions

(14) $$J=\lambda_{i}\lambda_{j}\lambda_{k}=1,\;\;P_{jj}=0,\;\;P_{kk}=0.$$

The objective function for parameter identification is defined as the equally weighted average of the mean squared residuals between the predicted and experimental nominal stresses in the warp and weft directions

(15) $$f(\beta )=\frac{1}{2}\left[\frac{1}{n_{1}}\sum_{r=1}^{n_{1}}{\left(P_{11}^{\mathrm{pred}}(\lambda_{1,r};\beta )-P_{11,r}^{\exp}\right)}^{2}+\frac{1}{n_{2}}\sum_{s=1}^{n_{2}}{\left(P_{22}^{\mathrm{pred}}(\lambda_{2,s};\beta )-P_{22,s}^{\exp}\right)}^{2}\right].$$

where β is the vector of parameters to be identified, $n_{1}$ and $n_{2}$ are the numbers of valid warp and weft data points, respectively, and subscripts 1 and 2 denote the warp and weft directions.

The objective function is nonlinear in multiple material parameters and may exhibit local minima. Therefore, a purely local search result may depend on the initial parameter vector. Accordingly, a hybrid identification strategy is adopted, in which simulated annealing performs the global search and gradient descent refines the best current solution [42,43]. During simulated annealing, a new parameter vector $\beta^{\prime }$ is generated by a random perturbation of the current vector β. The resulting objective increment is then evaluated

(16) $$\Delta f=f(\beta^{\prime })-f(\beta ).$$

The vector that decreases the objective function is accepted directly. Otherwise, it is accepted according to the Metropolis probability criterion

(17) $$r<\exp(-\Delta f/T_{k}),\;\;\;r\in [0,1].$$

The annealing temperature is updated according to the prescribed cooling relation

(18) $$T_{k+1}=\theta_{k}T_{k}.$$

where k is the simulated annealing iteration number and $\theta_{k}=\exp\left(-0.02k^{1/10}\right)$ is the corresponding cooling coefficient.

**Figure 5:**
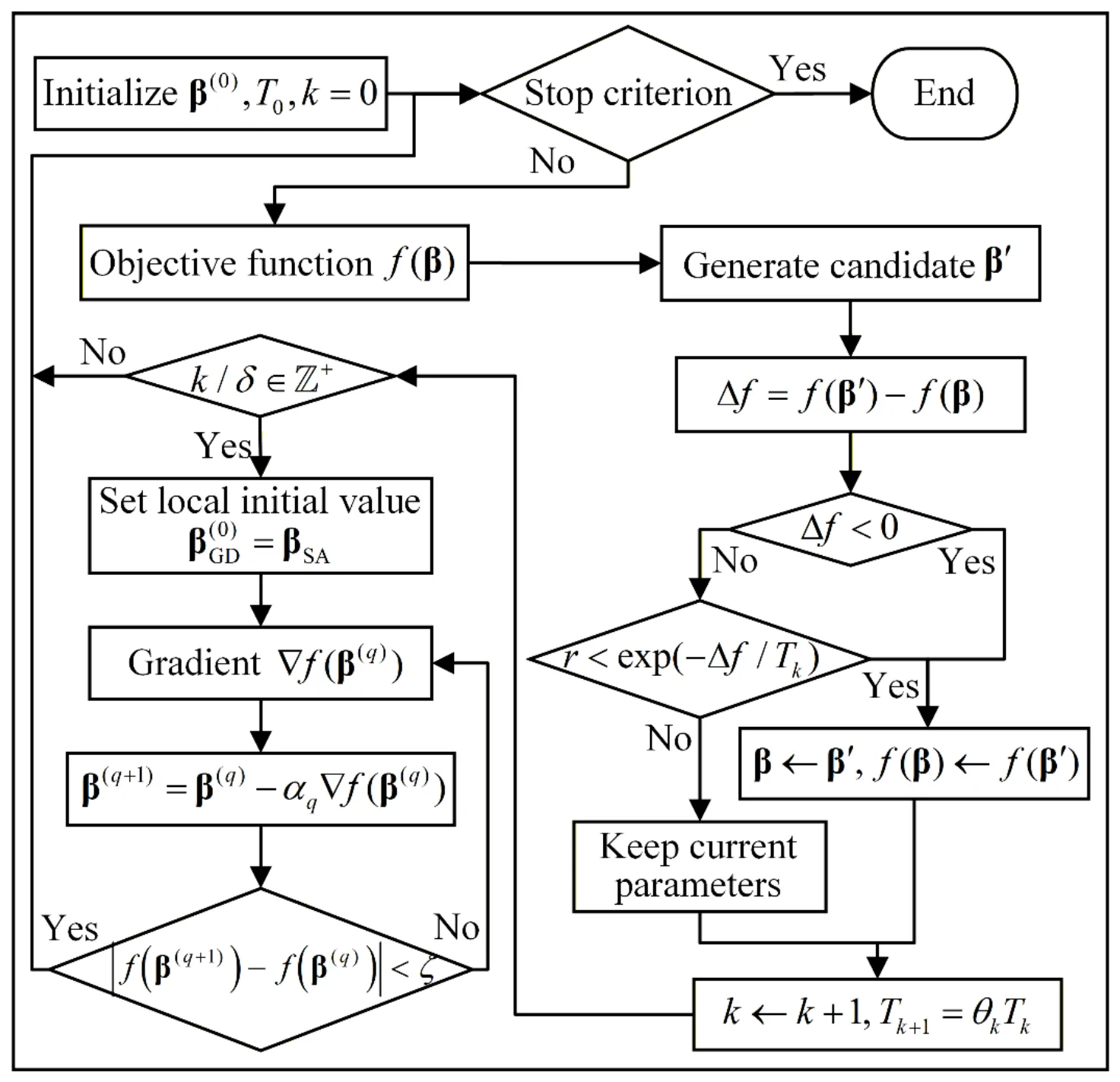
Hybrid procedure for parameter identification combining simulated annealing and gradient descent.

After every prescribed block of annealing iterations δ=100, the best parameter vector obtained at that stage is used as the initial value for gradient-descent refinement, as shown in Figure 5.

A local update is retained only when it decreases the objective function, and the local update is performed as

(19) $$\beta^{\left(q+1\right)}=\beta^{\left(q\right)}-\alpha_{q}\nabla f\;\left(\beta^{\left(q\right)}\right).$$

where q is the gradient descent iteration number, and the initial learning rate is $\alpha_{0}=0.01$.

When the local stopping condition is reached, the simulated annealing search is resumed

(20) $$\left|f\left(\beta^{\left(q+1\right)}\right)-f\;\left(\beta^{\left(q\right)}\right)\right|<\zeta ,\;\;\zeta =0.005\;{\mathrm{MPa}}^{2}.$$

The parameter-identification procedure terminates when the number of simulated annealing iterations reaches $k_{\max}=10000$ or the best objective-function value satisfies $f_{\mathrm{best}}\leq 1\;{\mathrm{MPa}}^{2}$.

**Figure 6:**
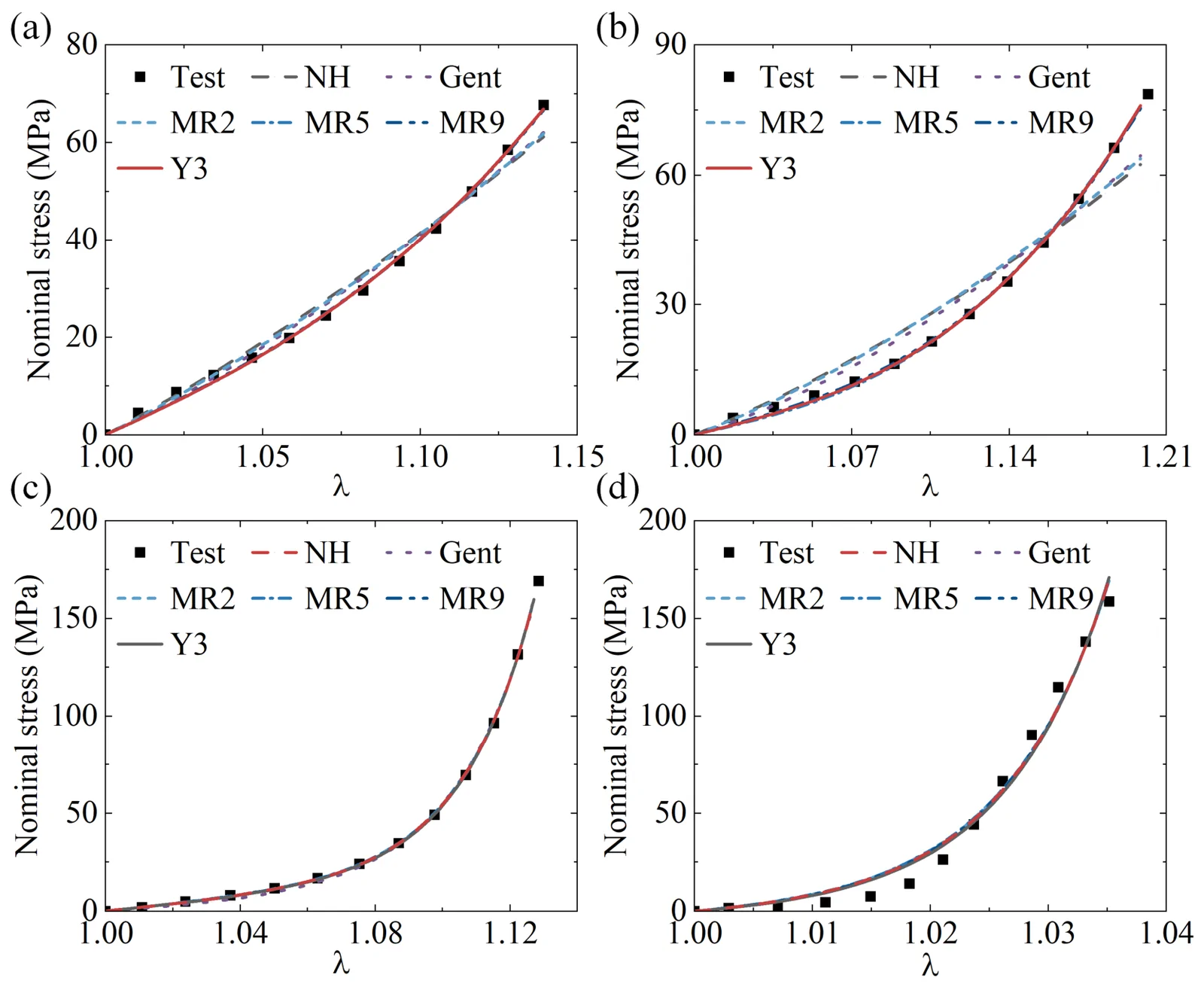
Comparison between the parameter-identification results of the six model combinations with the average uniaxial tensile curves: (**a**) TPU in the warp direction; (**b**) TPU in the weft direction; (**c**) UHMWPE in the warp direction; and (**d**) UHMWPE in the weft direction.

**Figure 7:**
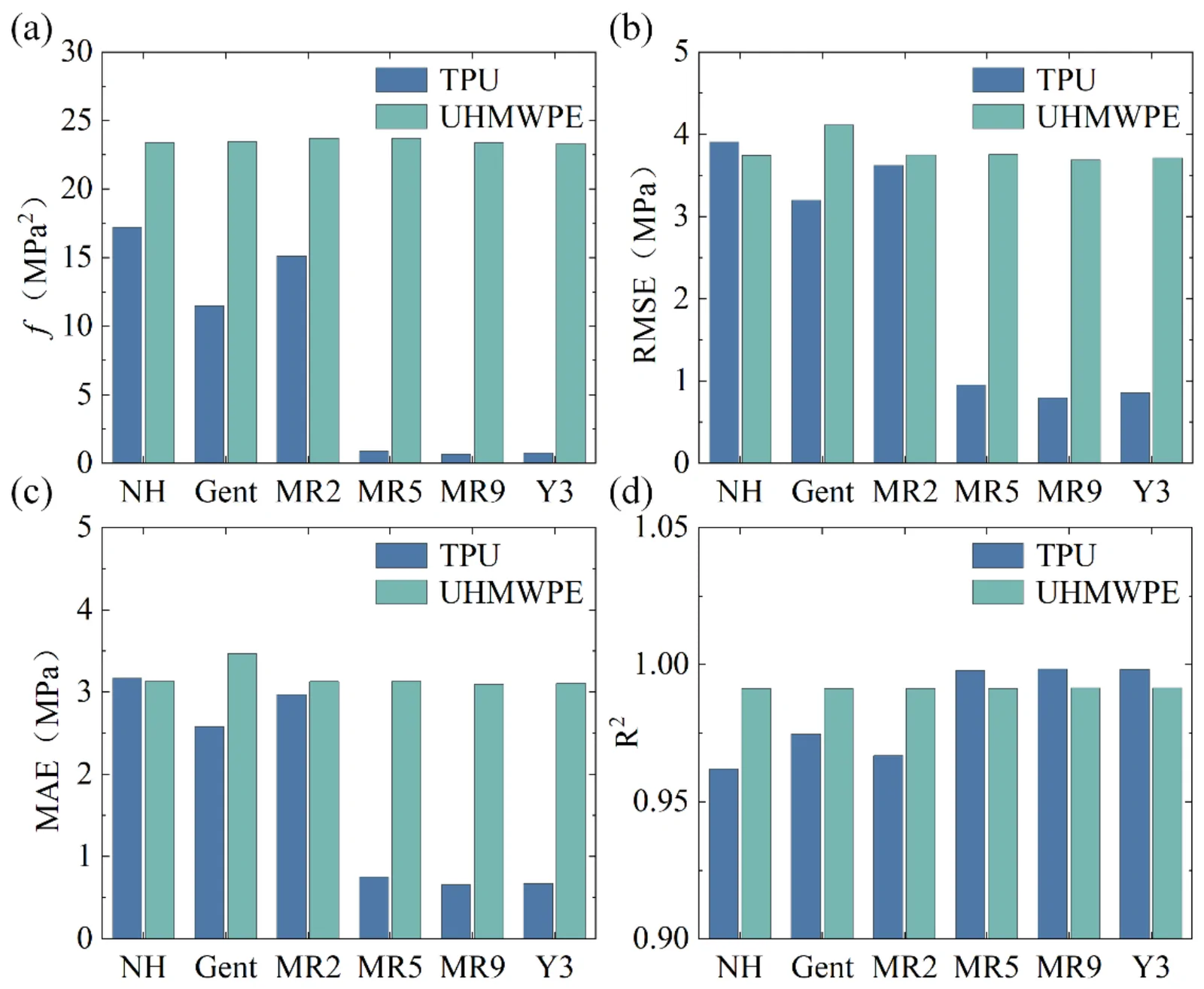
Comparison of the parameter-identification metrics of the six model combinations: (**a**) objective function value f; (**b**) average RMSE; (**c**) average MAE; and (**d**) average R^2^.

In addition to the objective function, the root-mean-square error (RMSE), mean absolute error (MAE), and coefficient of determination R^2^ are used to evaluate the parameter-identification results for both materials

(21) $$\begin{array}{l} \mathrm{RMSE}=\sqrt{\frac{1}{N}\sum_{i=1}^{N}{\left(P_{i}^{\mathrm{pre}}-P_{i}^{\exp}\right)}^{2}},\\ \mathrm{MAE}=\frac{1}{N}\sum_{i=1}^{N}\left|P_{i}^{\mathrm{pre}}-P_{i}^{\exp}\right|,\\ R^{2}=1-\frac{\sum_{i=1}^{N}{\left(P_{i}^{\exp}-P_{i}^{\mathrm{pre}}\right)}^{2}}{\sum_{i=1}^{N}{\left(P_{i}^{\exp}-{\bar{P}}^{\exp}\right)}^{2}}. \end{array}$$

where $P_{i}^{\mathrm{pre}}$ and $P_{i}^{\exp}$ are the predicted and experimental nominal stresses, respectively, N is the number of valid data points in the corresponding direction, and ${\bar{P}}^{\exp}$ is the average experimental nominal stress. The RMSE, MAE, and R^2^ are calculated separately for the warp and weft directions. Figure 6 compares the parameter-identification results of the six model combinations with the average experimental curves, while the corresponding average evaluation values are presented in Figure 7.

With the same weak orthotropic correction, the NH, Gent, and MR2 combinations do not adequately capture the progressive stiffening of TPU at intermediate and high stretches, yielding objective-function values of 17.2148, 11.4837, and 15.1288 MPa^2^, respectively. In contrast, the corresponding values for MR5, MR9, and Y3 decrease to 0.9014, 0.6476, and 0.7381 MPa^2^. MR9 yields the lowest objective-function value; however, its average R^2^ differs from that of Y3 by a negligible margin of 0.0002, and the corresponding curves are nearly coincident over the parameter-identification range. Given the essentially equivalent identification accuracy of the two models, Y3 with fewer isotropic parameters is selected as the isotropic base for the weak orthotropic model of TPU.

For UHMWPE, the six model combinations produce only minor differences in the predicted responses. The objective-function values range from 23.2846 to 23.6966 MPa^2^, with a maximum difference of only 0.4120 MPa^2^. All average R^2^ values are greater than 0.99 and differ by no more than 0.0001. These results show that the predicted response of UHMWPE is dominated by the strong orthotropic correction, with only a limited influence from the specific form of the isotropic basis. The NH model combined with the strong orthotropic correction is therefore adopted for UHMWPE. The selected model gives average RMSE, MAE, and R^2^ values of 3.7447 MPa, 3.1269 MPa, and 0.9913, respectively.

Accordingly, the resulting strain-energy functions adopted for TPU and UHMWPE are

(22) $$W_{\mathrm{TPU}}=W_{\mathrm{iso}}^{Y3}+W_{\mathrm{ani}}^{w},\;\;W_{\mathrm{UHMWPE}}=W_{\mathrm{iso}}^{\mathrm{NH}}+W_{\mathrm{ani}}^{s}.$$

The corresponding identified parameter sets adopted for the present model are listed in Table 1 and Table 2. Although several $k_{ij}$ values are negative, these parameters represent residual directional stiffness corrections relative to an isotropic reference rather than independent elastic moduli. A numerical stability check is conducted on the strain energy function of the TPU within the deformation range investigated in this study. The constrained tangent and incremental responses remained positive at all evaluated states and directions, with no stress reversal, negative incremental stiffness, or non-physical response identified. Moreover, an additional local sensitivity check is conducted by perturbing each identified parameter individually by ±5% while retaining the other parameters in both directions. The results show significant non-uniform sensitivities. The main influencing factors for TPU are $C_{10}$, $C_{20}$, and $k_{22}$, whereas $C_{30}$, $k_{12}$, and $k_{66}$ have limited impact. For UHMWPE, the directional parameters $A_{1}$ and $A_{2}$ produce the largest changes, followed by $D_{1}$ and $D_{2}$. These results are consistent with the strong orthotropic formulation, in which the directional terms dominate the marked differences in warp and weft responses. Notably, the identified parameters listed in the tables are the optimal values under the prescribed iterative times, rather than strict global unique parameter vectors.

### 2.4. Numerical Implementation and Verification

The developed anisotropic hyperelastic models for TPU and UHMWPE are implemented in Abaqus/Explicit 2020 through VUANISOHYPER_INV. The subroutine evaluates the strain-energy function in terms of the relevant invariants and returns its first- and second-order derivatives to the solver.

Based on the specimen dimensions specified in Section 2.1, finite element models are constructed for both materials in the warp and weft directions. The specimens are discretized using S4R shell elements with a mesh size of 2 mm, and the shell thickness is set to the measured average value for each material. The gripping regions at both ends are coupled to reference points, and an axial displacement corresponding to the stretch range used for parameter identification is prescribed. The local material directions 1 and 2 are aligned with the loading direction in the warp and weft models, respectively. The finite-element results are processed using the same definitions as in the experiments and compared with the model predictions.

**Figure 8:**
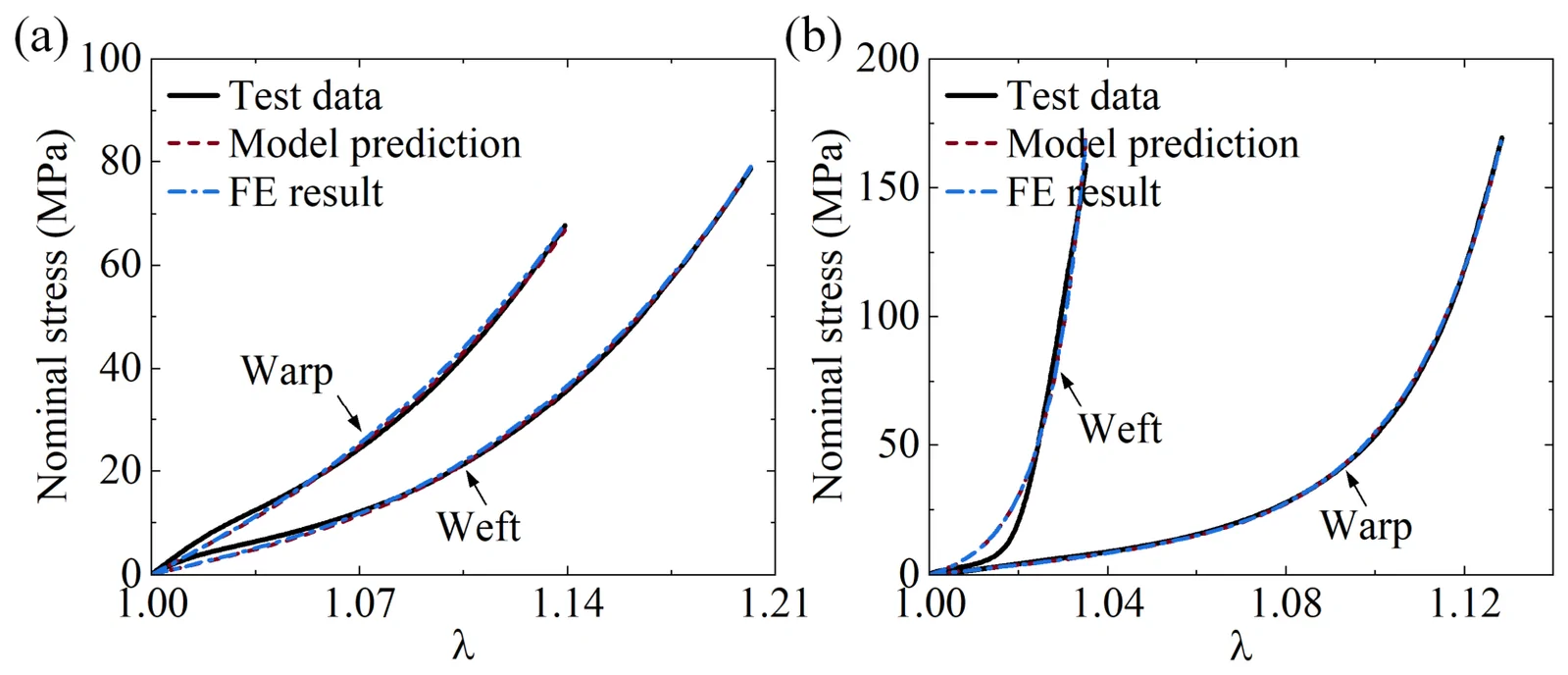
Comparison of the test data, model predictions, and finite-element results for the uniaxial tensile responses: (**a**) TPU and (**b**) UHMWPE.

A mesh-convergence assessment is additionally performed with mesh sizes of 1 mm and 4 mm. Compared with 1 mm results, the maximum difference in stress across the four material directions is less than 0.2%, indicating that the effect of further refinement on the response is negligible. As shown in Figure 8, the finite element results are in good agreement with the model predictions for both materials and in both warp and weft directions and reproduce the nonlinear response trends observed experimentally. These results confirm that the numerical implementation is consistent with the anisotropic hyperelastic models and provide a basis for subsequent analysis of the flexible anti-collision airbag.

## 3. Quasi-Static Compression Tests and Numerical Validation

Localized quasi-static compression tests are conducted to characterize the mechanical response of the multilayer airbag and to examine the effects of initial internal pressure and loading rate. A corresponding finite element model is subsequently developed using the anisotropic hyperelastic models established in Section 2 and validated against the test.

### 3.1. Experimental Setup

**Figure 9:**
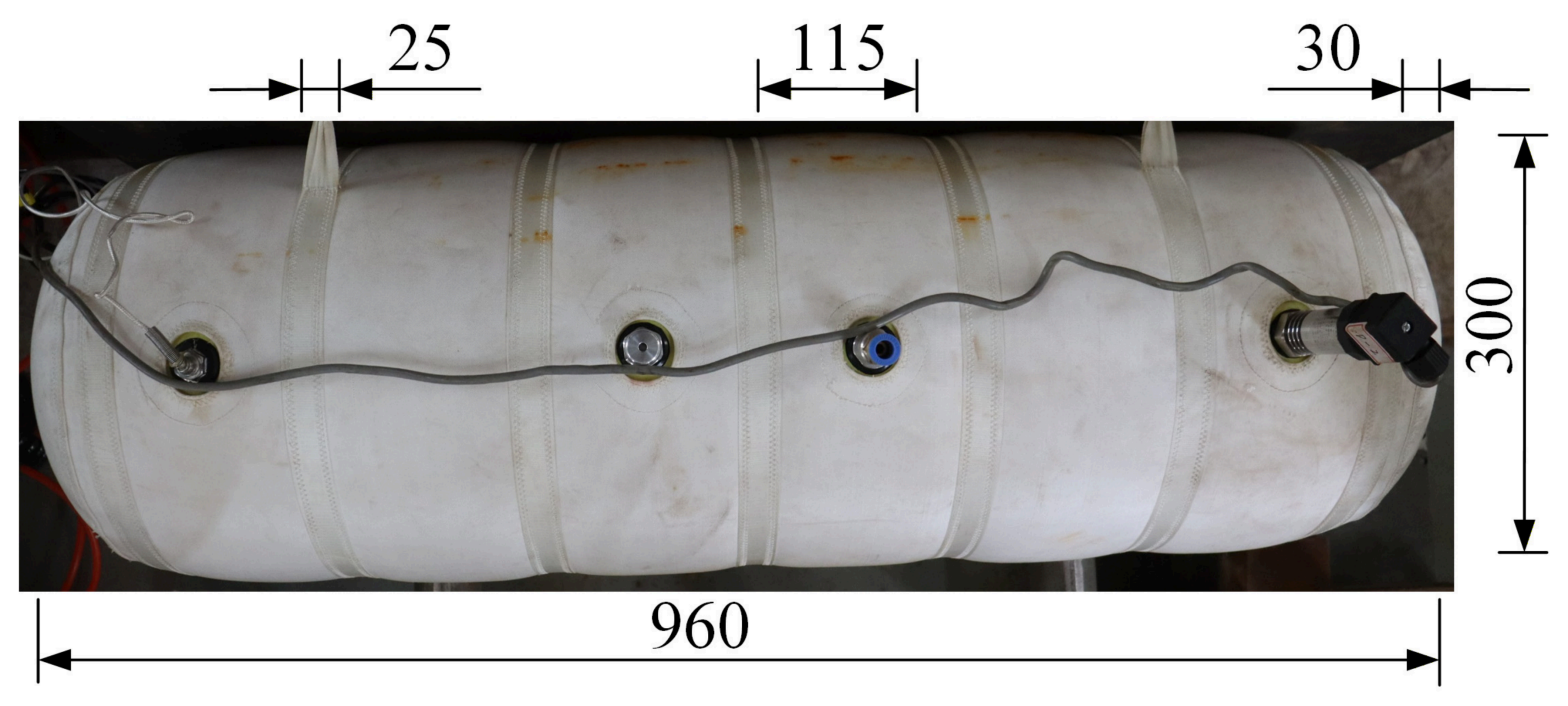
Main dimensions of the airbag (mm).

The airbag specimen is fabricated by the manufacturer according to the prescribed dimensions. As shown in Figure 9, the single-chamber specimen has a total length of 960 mm and comprises a 900 mm cylindrical section with a diameter of 300 mm and two end sections with a maximum axial height of 30 mm. The continuous airbag layer consists of a TPU inner layer forming an airtight layer and two woven UHMWPE reinforcing layers characterized in Section 2. These three continuous layers are not directly bonded together; instead, seven polyamide webbings are placed at equal intervals along the axial direction and stitched around the circumference, thereby locally connecting each webbing to all three layers. Each webbing has a width of 25 mm and a thickness of 0.70 mm. The webbings are considered local restraining components and are not counted as an additional continuous layer. In addition, the airbag is equipped with straps at the second and sixth webbing positions to secure the specimen during testing. Four ports are incorporated for inflation, pressure relief, and measurement of internal pressure and gas temperature.

**Figure 10:**
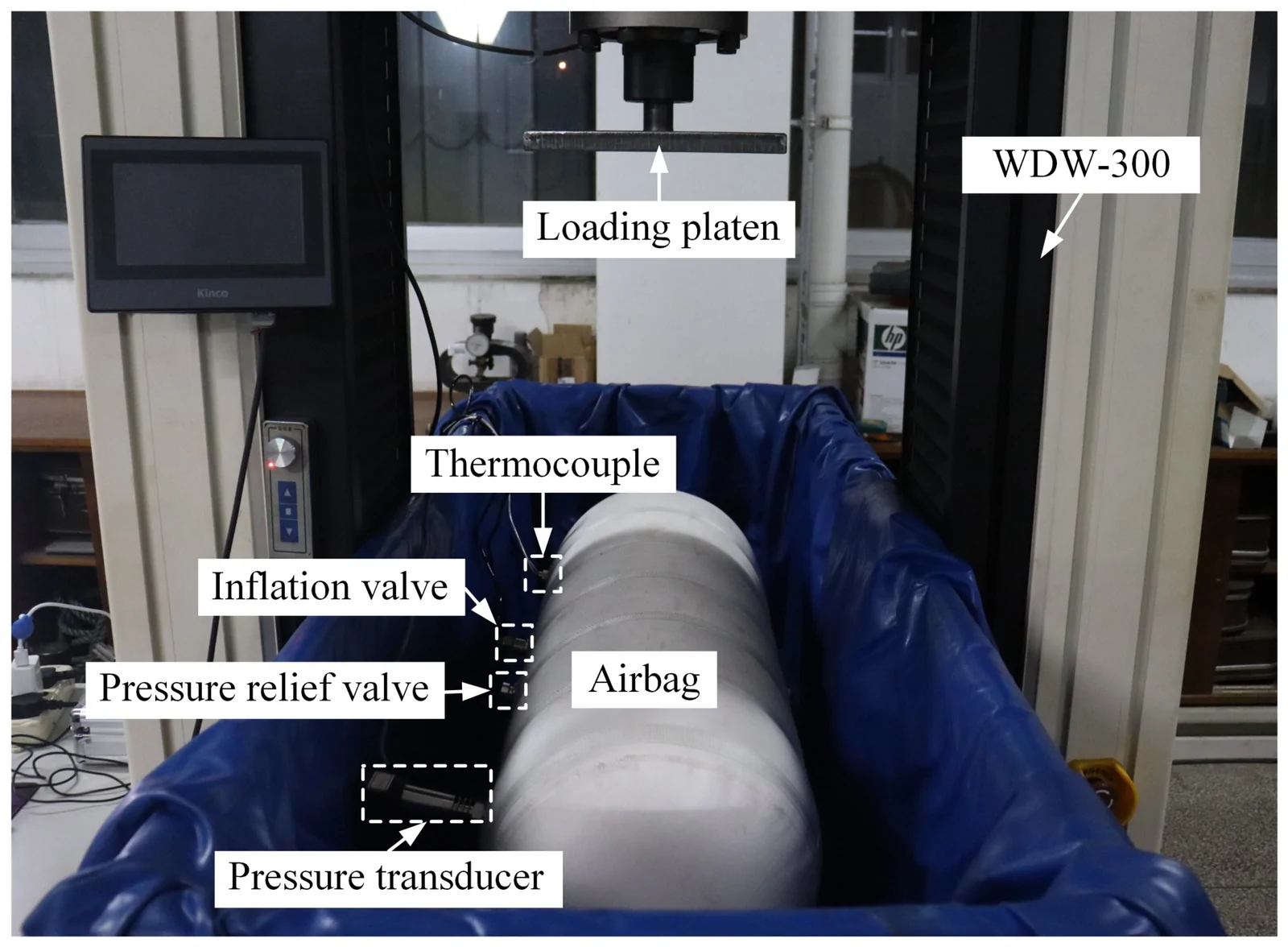
Experimental setup for localized quasi-static compression of the flexible airbag.

The specimen is placed horizontally between a square loading platen and the rear measuring assembly of a WDW-300 electronic universal testing machine (Changchun Kexin Experimental Instrument Co., Ltd., Changchun, China), as shown in Figure 10. The loading platen has a contact surface of 250 mm × 250 mm with a thickness of 20 mm, and applies vertical compression at the axial centre of the cylindrical section. The rear measuring assembly consists of a measuring truss of 1.0 m × 0.3 m and a rigid support plate of 1.35 m × 0.35 m. Two straps are attached to the support plate to hold the specimen during loading.

Three load cells are installed between the measurement truss and support plate in a triangular arrangement. The rear-side force F is obtained by summing the three measured components. A pressure transducer and temperature sensor record internal pressure and gas temperature, respectively. All signals are acquired simultaneously using a DH5902N data acquisition system (Jiangsu Donghua Testing Technology Co., Ltd., Jingjiang, China), and all sensors are calibrated before testing. It should be noted that the apparatus is installed in a water tank equipped with a waterproof liner, which is provided for a separate series of tests involving water. However, all the tests investigated in this study are conducted under dry conditions, and the airbag remains out of contact with the liner throughout loading.

The test cases with initial internal pressures $p_{0}$ of 0.12, 0.13, and 0.14 MPa are conducted at a loading rate of 5 mm/min. To examine the effect of loading rate, an additional test is performed at 50 mm/min with an initial pressure of 0.12 MPa. The compression ratio is defined as $\eta =\delta /D_{0}$, where δ is the loading platen displacement and the airbag diameter $D_{0}$ is 300 mm. The tests are terminated at δ=210 mm, corresponding to η=0.7. All tests are conducted at an ambient temperature of 293.15 K and an atmospheric pressure of 0.101 MPa. An automatic pressure-relief valve with a venting threshold of 0.15 MPa is used in all tests. Each condition is repeated three times, and the averaged responses are used in the subsequent analysis.

### 3.2. Experimental Results

**Figure 11:**
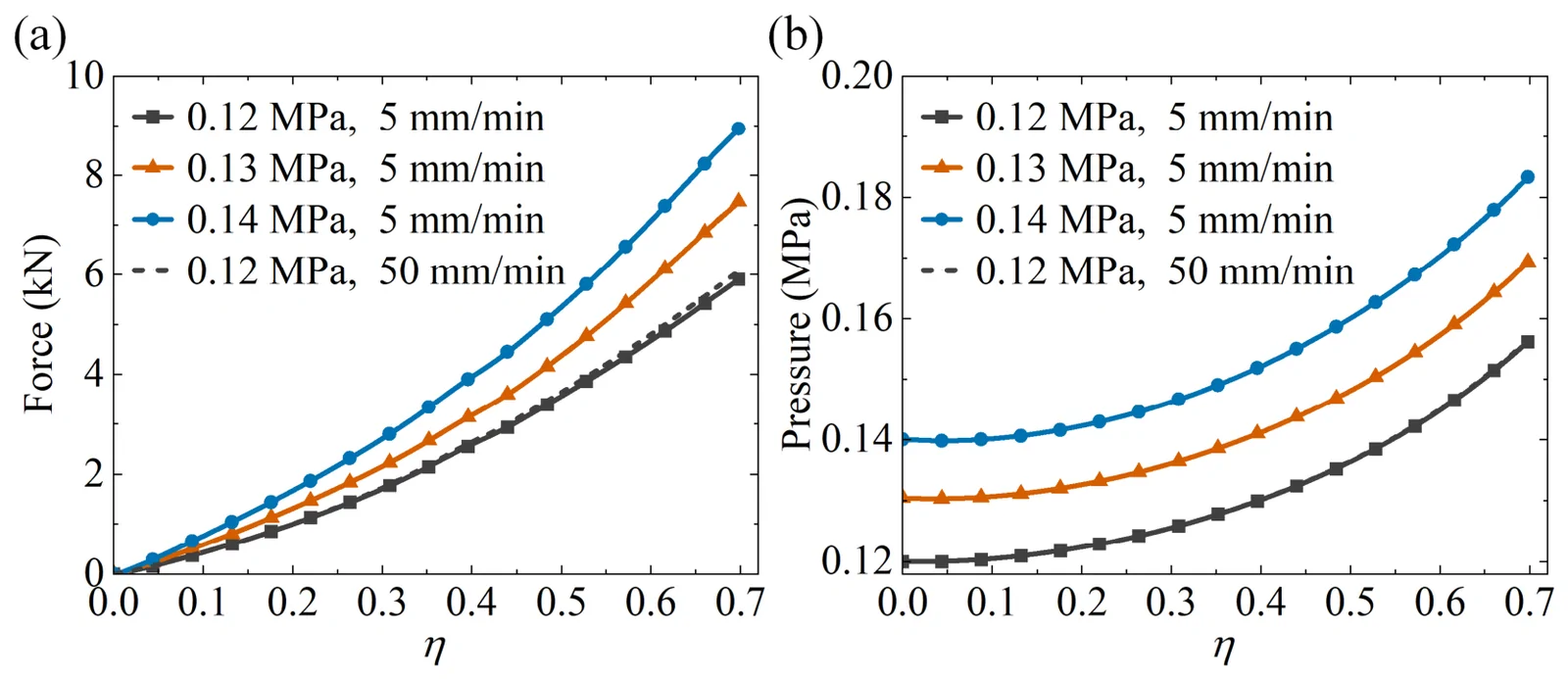
Experimental responses under different initial internal pressures and loading rates: (**a**) rear-side force; (**b**) internal pressure.

Figure 11 shows the averaged rear-side force F and internal pressure p for all four test conditions. For all test conditions, F increases monotonically with compression ratio η. The slopes increase progressively with η, indicating a continuous increase in compressive stiffness. No abrupt force reduction or visible damage is observed up to the maximum compression ratio of η=0.7. The pressure curves show similar upward curvature.

At the fixed loading rate of 5 mm/min, the initial pressure has a significant effect on the force response. Increasing $p_{0}$ shifts the entire curve upward, and the separation among the curves becomes greater at larger compression ratios. At η=0.7, the measured rear-side forces are 5.921 ± 0.082, 7.484 ± 0.129, and 8.948 ± 0.075 kN for $p_{0}$ values of 0.12, 0.13, and 0.14 MPa, respectively. The pressures are 0.15630 ± 0.00015, 0.16945 ± 0.00013, and 0.18344 ± 0.00008 MPa, corresponding to mean pressure increments of 0.03630, 0.03945, and 0.04344 MPa. Since the reduction in airbag volume and the limited capacity of the gas discharge occur simultaneously, the measured maximum pressure is significantly higher than 0.15 MPa. Thus, increasing $p_{0}$ from 0.12 to 0.14 MPa increases the force by about 51%, demonstrating the strong dependence of the compressive resistance on the initial pressure. Compared with the 0.12 MPa case, pressure increments for the 0.13 and 0.14 MPa cases increase by only 8.68% and 19.68%, respectively. Although the pressure remains higher at larger $p_{0}$, the three curves exhibit similar evolution throughout compression.

At $p_{0}$ of 0.12 MPa, increasing the loading rate from 5 to 50 mm/min produces only a slight increase in the rear-side force, while the corresponding pressure curves are nearly coincident. At η=0.7, the rear-side force increases from 5.921 ± 0.082 to 6.088 ± 0.037 kN, a difference of 2.81%, while the pressure changes from 0.15630 ± 0.00015 to 0.15651 ± 0.00008 MPa, and the corresponding pressure increment increases by 0.58%. The response curves remain closely aligned throughout compression, indicating that increasing the prescribed loading rate to 50 mm/min produces only a negligible change in the measured compression response. For the present specimen and test configuration, 50 mm/min can therefore still be regarded as a quasi-static loading rate.

### 3.3. Numerical Validation

The finite element results are compared with the experimental results to validate the model. The parametric analysis is then performed based on the validated model to investigate the effects of airbag geometry, loading platen width, and axial eccentricity on the cushioning performance of the airbag.

**Figure 12:**
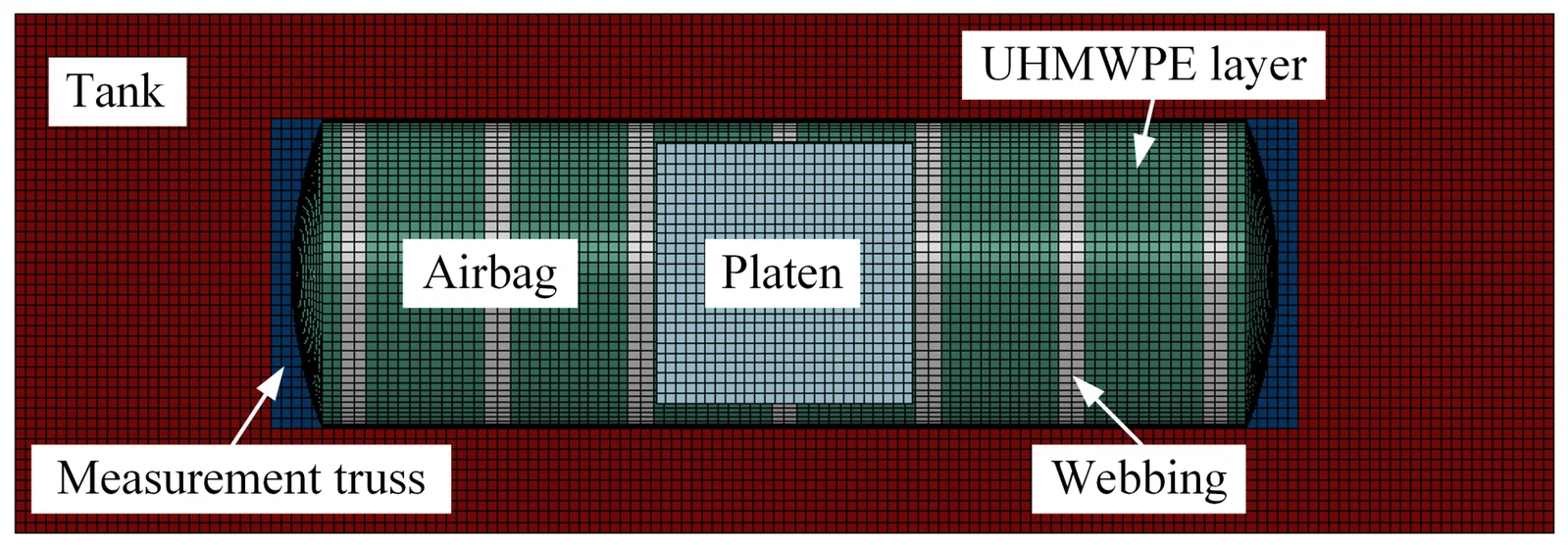
Top view of the finite element model for the localized quasi-static compression test.

The finite element model of the test case with an initial internal pressure of 0.12 MPa and a loading rate of 5 mm/min is developed in Abaqus/Explicit, as shown in Figure 12. The interaction topology follows the physical multilayer construction. The inner TPU layer, the two outer UHMWPE fabric layers, and the webbing are all represented by independent shell components with S4R elements. Away from the stitched regions, the adjacent layers interact through surface-based general contact rather than adhesive constraints. The contact definitions are also applied between the outer airbag or webbing surface and the loading platen and rear measurement components, and the inner airbag surface incorporates self-contact to prevent interpenetration, with a coefficient of friction of 0.20. At each webbing location, tie constraints connect the webbing and all three layers to represent the local stitched attachment. The polyamide webbings are modelled as linear elastic, with a Young’s modulus of 1.0 GPa and a Poisson’s ratio of 0.30. These values are engineering estimates provided by the airbag manufacturer. The webbings occupy discrete 25-mm-wide stitched strips, primarily connecting the three continuous material layers locally and restricting relative movement at the stitched positions. Accordingly, the independently validated prediction of local webbing is not the focus of the present study.

The airbag is defined as a fluid cavity with an initial absolute pressure and gas temperature of 0.12 MPa and 293.15 K, respectively. The venting process and the associated reduction in cavity gas mass are represented by the fluid-exchange interaction from the cavity to the environment, with an effective exchange area of 0.000219 m^2^. The loading platen and rear measurement assembly are treated as rigid bodies. During compression, the rigid loading platen applies the prescribed displacement to the exposed outer UHMWPE surface and the webbings within the loaded region. The surrounding tank is retained to reproduce the experimental arrangement but remains out of contact with the airbag. As no visible damage or material failure occurs within the investigated compression range, no damage or failure criteria are included.

**Figure 13:**
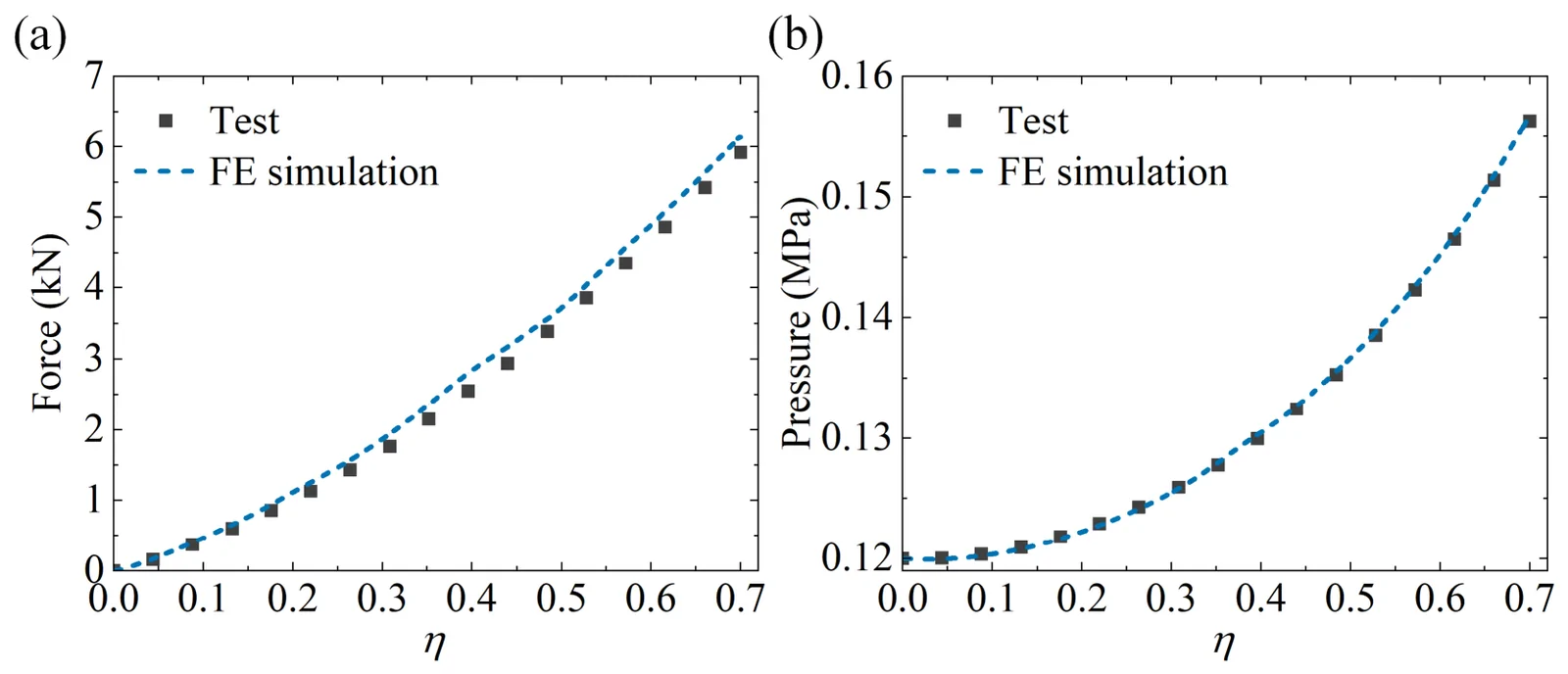
Comparison of the experimental and finite element responses for 0.12 MPa and 5 mm/min: (**a**) rear-side force; (**b**) internal pressure.

As shown in Figure 13, the numerical results capture the monotonic nonlinear increases in both the rear-side force and internal pressure of the airbag. The predicted force is slightly higher than the experimental result over the intermediate and large compression ranges, while the pressure curves are nearly coincident throughout loading. At η=0.7, the numerical force exceeds the experimental value by 0.2138 kN, corresponding to a relative difference of 3.61%. The experimental force data show excellent agreement with the numerical results, with an R^2^ value of 0.9906.

The pressure response yields an R^2^ value of 0.9995 over the entire compression range and a relative difference of only 0.228% at η=0.7. The predictive capability of the finite element model is further assessed against the remaining experimental cases, as shown in Table 3. Within the range of 0≤η≤0.7, the R^2^ values for force range from 0.9854 to 0.9932, while those for pressure range from 0.9995 to 0.9999. At η=0.7, the corresponding relative differences are less than 5% for force and 1% for pressure. The close agreement in both the force and pressure responses further supports the applicability of the anisotropic hyperelastic material models developed in Section 2 and demonstrates that the finite element model captures the coupled structural deformation and cavity pressurization under localized quasi-static compression. The validated model is therefore adopted for the subsequent parametric analysis.

## 4. Parametric Analysis

In this section, the validated finite element model is used to investigate the effects of the prescribed loading rate v, axial loading length ratio $\eta_{L}$, airbag aspect ratio Λ, and axial eccentricity $\eta_{e}$ on the terminal compression response of the airbag at η=0.7. Within the compression range, no visible damage is observed in the experiments. Both force and pressure rise monotonically and steadily, and stiffness also gradually increases. Accordingly, all analyses adopt an initial pressure $p_{0}$ of 0.12 MPa, and the response is characterized by the rear-side force $F_{\eta =0.7}$ and the pressure increment $\Delta p_{\eta =0.7}=p_{\eta =0.7}-p_{0}$.

### 4.1. Effect of Loading Rate

**Figure 14:**
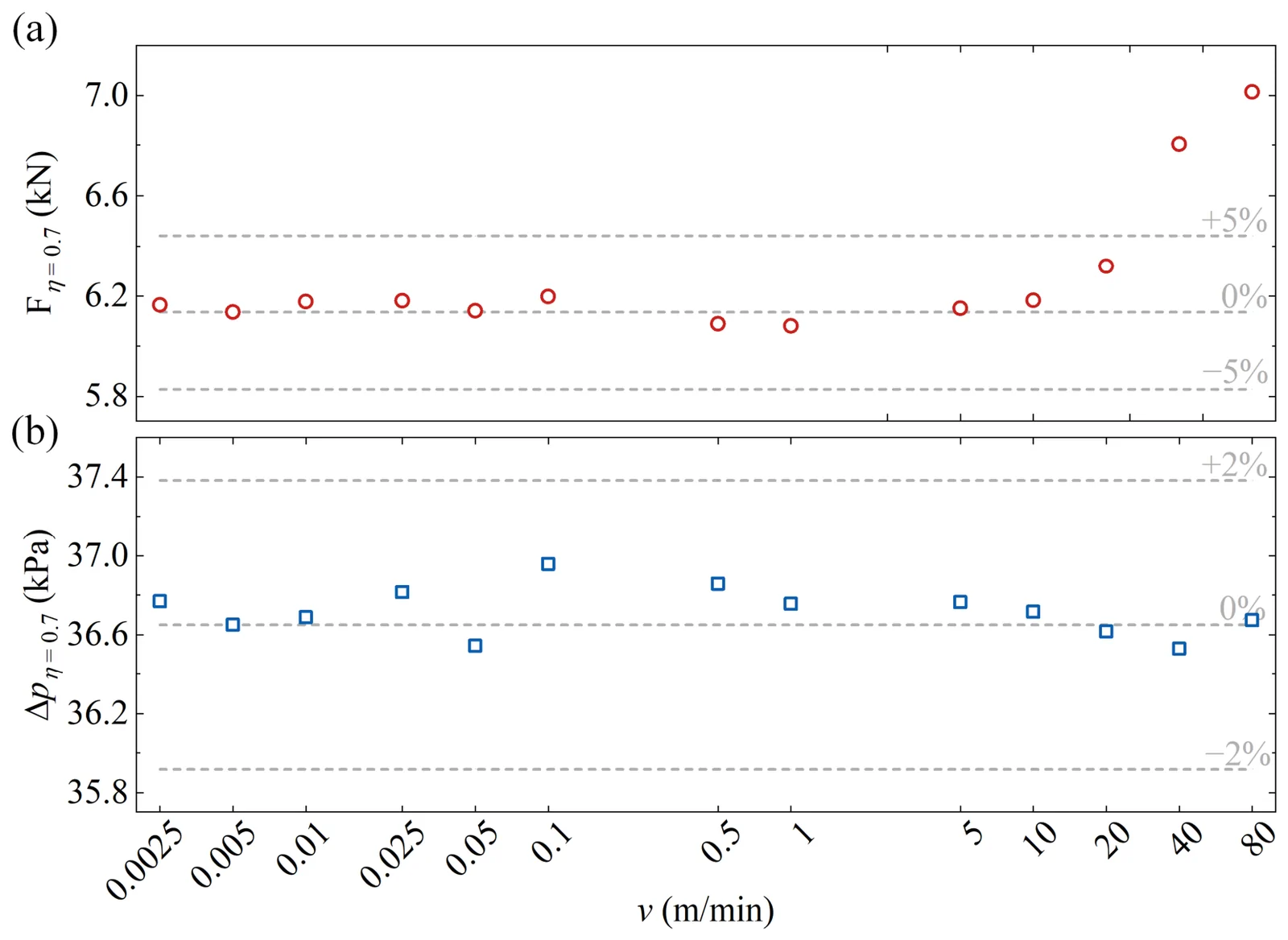
Effect of the prescribed loading rate on the terminal compression response at η=0.7: (**a**) rear-side force; (**b**) pressure increment.

To examine the effect of the prescribed loading rate, thirteen values, 0.0025, 0.005, 0.010, 0.025, 0.050, 0.100, 0.500, 1, 5, 10, 20, 40, and 80 m/min, are considered. The 0.005 m/min case is used as the numerical reference. The results are presented in Figure 14, where the dashed lines denote deviations of ±5% for the force and ±2% for the pressure increment.

As shown in Figure 14a, $F_{\eta =0.7}$ remains stable over a wide range of loading rates. Between 0.0025 and 10 m/min, the force varies from 6.081 to 6.198 kN, corresponding to deviations from 0.88% to 1.02% with respect to the reference. Increasing the loading rate to 20 m/min raises the force to 6.319 kN, a deviation of 3.00%, remaining within 5%. A pronounced increase occurs at 40 and 80 m/min, where $F_{\eta =0.7}$ reaches 6.803 and 7.015 kN, corresponding to increases of 10.89% and 14.34%, respectively.

Across all 13 cases, $\Delta p_{\eta =0.7}$ remains between 36.530 and 36.957 kPa, with corresponding deviations ranging from 0.33% to 0.84%, and no monotonic variation with v is observed. In contrast to the significant increase in force at the two highest rates, the pressure increment remains close to the reference value. $\Delta p_{\eta =0.7}$ is therefore essentially independent of loading rate over the investigated range.

The force and pressure responses diverge at the higher prescribed loading rates. Relative to the 0.005 m/min reference case, the force deviation remains within approximately 1% for prescribed loading rates up to 10 m/min. The force begins to deviate at 20 m/min and exhibits pronounced amplification of 10.89% and 14.34% at 40 and 80 m/min, respectively. In contrast, $\Delta p_{\eta =0.7}$ varies by less than 1% over the investigated range. Accordingly, 10 m/min can be used as an approximate numerical quasi-static limit for the current model. Since the present constitutive models are rate-independent, the force amplification without a corresponding pressure rise indicates the growing influence of structural inertia.

### 4.2. Effect of Axial Loading Length Ratio

The axial loading length ratio is defined as $\eta_{L}=L_{P}/L_{0}$, where $L_{P}$ is the axial length of the loading platen and $L_{0}$ is the length of the cylindrical section. The cylindrical-section length $L_{0}$ and airbag diameter $D_{0}$ are fixed at 0.90 m and 0.30 m, respectively, and the loading rate v is 0.005 m/min, while $\eta_{L}$ varies from 0.2 to 0.6. The corresponding values of $L_{P}$ are 0.18, 0.27, 0.36, 0.45, and 0.54 m. The width of the platen $B_{P}$ is maintained at 0.25 m.

**Figure 15:**
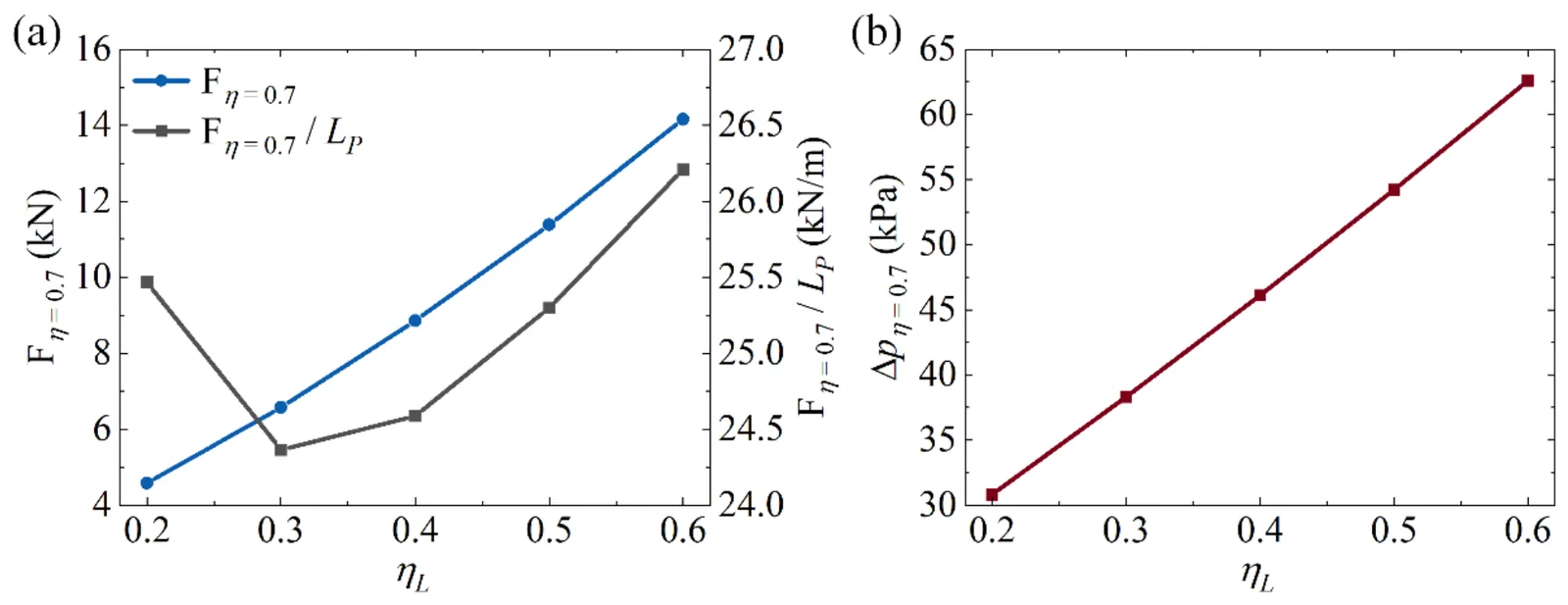
Effect of axial loading length ratio on the terminal compression response at η=0.7: (**a**) rear-side force and force per unit axial loading length; (**b**) pressure increment.

Figure 15a shows that $F_{\eta =0.7}$ increases monotonically with $\eta_{L}$. As $\eta_{L}$ increases from 0.2 to 0.6, the force rises from 4.584 to 14.152 kN, corresponding to an increase of 208.7%. The slope increases slightly with $\eta_{L}$, indicating that increasing $L_{P}$ enlarges the axial extent subjected to transverse compression, thereby allowing a greater region of the pressurized airbag to contribute to the rear-side force.

However, $F_{\eta =0.7}/L_{P}$ remains within a narrow range of 24.36–26.21 kN/m, decreasing slightly from 25.467 kN/m at $\eta_{L}=0.2$ to a minimum of 24.364 kN/m at $\eta_{L}=0.3$, and then increases gradually with further increases in $\eta_{L}$. The maximum variation in $F_{\eta =0.7}/L_{P}$ is only 7.56%, whereas $F_{\eta =0.7}$ increases more than three times. Therefore, the dominant effect of increasing $\eta_{L}$ is the enlargement of the loaded axial extent. Nevertheless, the deformation state and cavity pressure also change as the loading region expands, so the force does not scale strictly with $L_{P}$.

The pressure increment also increases monotonically with the axial loading length ratio. $\Delta p_{\eta =0.7}$ increases from 30.825 to 62.596 kPa as $\eta_{L}$ increases from 0.2 to 0.6, corresponding to an increase of 103.1%. The curve is approximately linear, with a slightly greater slope at larger values of $\eta_{L}$. At the same compression ratio η, a longer compressed region causes a greater reduction in cavity volume and thus a larger pressure increase. Consequently, increasing $\eta_{L}$ raises the force by enlarging the loaded region and increasing the internal pressure, while the effect on the force per unit axial loading length remains small.

### 4.3. Effect of Airbag Aspect Ratio

The airbag aspect ratio is defined as $\Lambda =L_{0}/D_{0}$, where the diameter $D_{0}$ is fixed at 0.30 m. Based on Section 4.2, the effect of the airbag aspect ratio Λ is examined under the constant axial loading length ratio at $\eta_{L}=0.4$. Accordingly, Λ varies from 2.0 to 3.6 by changing $L_{0}$ to 0.60, 0.72, 0.84, 0.96, and 1.08 m, with the corresponding $L_{P}$ values of 0.240, 0.288, 0.336, 0.384, and 0.432 m, respectively. The platen width $B_{P}$ is 0.25 m, and the loading rate v is 0.005 m/min.

**Figure 16:**
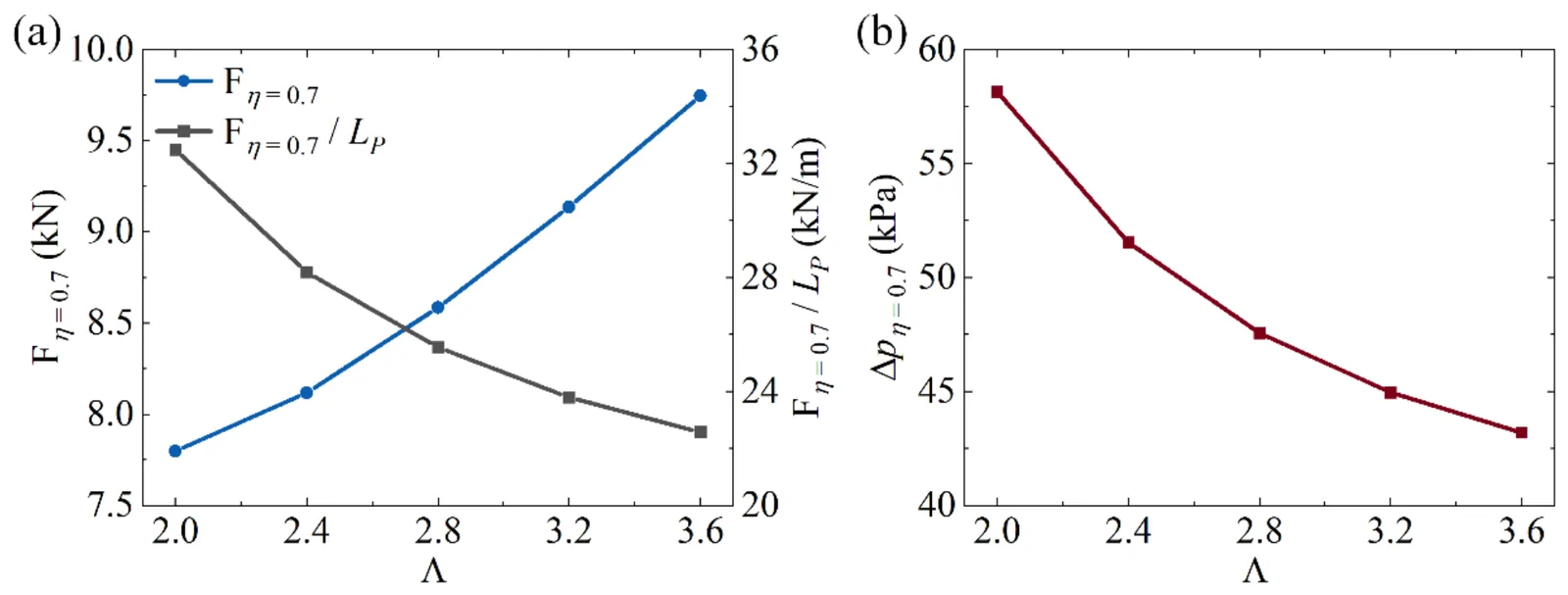
Effect of airbag aspect ratio on the terminal compression response at η=0.7 and $\eta_{L}=0.4$: (**a**) rear-side force and force per unit axial loading length; (**b**) pressure increment.

As shown in Figure 16a, $F_{\eta =0.7}$ increases monotonically with Λ, rising from 7.798 kN at Λ=2.0 to 9.746 kN at Λ=3.6, an increase of 25.0%. $F_{\eta =0.7}/L_{P}$ exhibits the opposite trend and decreases continuously from 32.49 to 22.56 kN/m, representing a reduction of 30.6%. The decrease in $F_{\eta =0.7}/L_{P}$ is more pronounced at lower aspect ratios and diminishes as Λ increases. Since $\eta_{L}$ is fixed at 0.4, $L_{P}$ increases by 80%. The increase in $F_{\eta =0.7}$ reflects the combined response of changes in airbag geometry and loading length, while the decrease in $F_{\eta =0.7}/L_{P}$ indicates a reduction in force per unit length.

The pressure increment decreases throughout the investigated range, as shown in Figure 16b. $\Delta p_{\eta =0.7}$ decreases from 58.157 kPa at Λ=2.0 to 43.185 kPa at Λ=3.6, corresponding to a reduction of 25.74%. The rate of decrease in the pressure increment becomes smaller at larger aspect ratios, closely following the trend of $F_{\eta =0.7}/L_{P}$. At constant $D_{0}$ and $\eta_{L}$, increasing $L_{0}$ lengthens both the loaded and unloaded regions of the airbag, so the deformation is accommodated over a greater axial extent and the relative reduction in cavity volume becomes smaller, leading to simultaneous decreases in $\Delta p_{\eta =0.7}$ and $F_{\eta =0.7}/L_{P}$. Nevertheless, the proportional increase in $L_{P}$ caused an increase in the total reaction force, thereby affecting the total force and the force per unit loading length in opposite directions.

### 4.4. Effect of Axial Eccentricity

The axial eccentricity ratio is defined as $\eta_{e}=e/L_{0}$, where e is the axial offset between the centre of the loading platen and the mid-length of the cylindrical section. The airbag length $L_{0}$ and diameter $D_{0}$ are 0.90 m and 0.30 m; the loading platen length $L_{P}$ and width $B_{P}$ are 0.36 m and 0.25 m, respectively, and the loading rate v is 0.005 m/min. Five eccentricity ratios $\eta_{e}$ of 0, 0.05, 0.10, 0.15, and 0.20 are considered, corresponding to axial offsets e of 0, 0.045, 0.090, 0.135, and 0.180 m, respectively. Since the airbag is symmetric, only one direction of axial offset is analyzed.

The rear-side force $F_{\eta =0.7}$ increases monotonically with $\eta_{e}$, rising from 8.851 kN under concentric loading to 12.882 kN at an eccentricity ratio of 0.20, which corresponds to an increase of 45.5%. The largest relative change occurs as $\eta_{e}$ increases from 0 to 0.05, with the force increasing by 20.67%. Thereafter, the force increases more gradually but continues to rise over the entire investigated range of eccentricity ratios.

**Figure 17:**
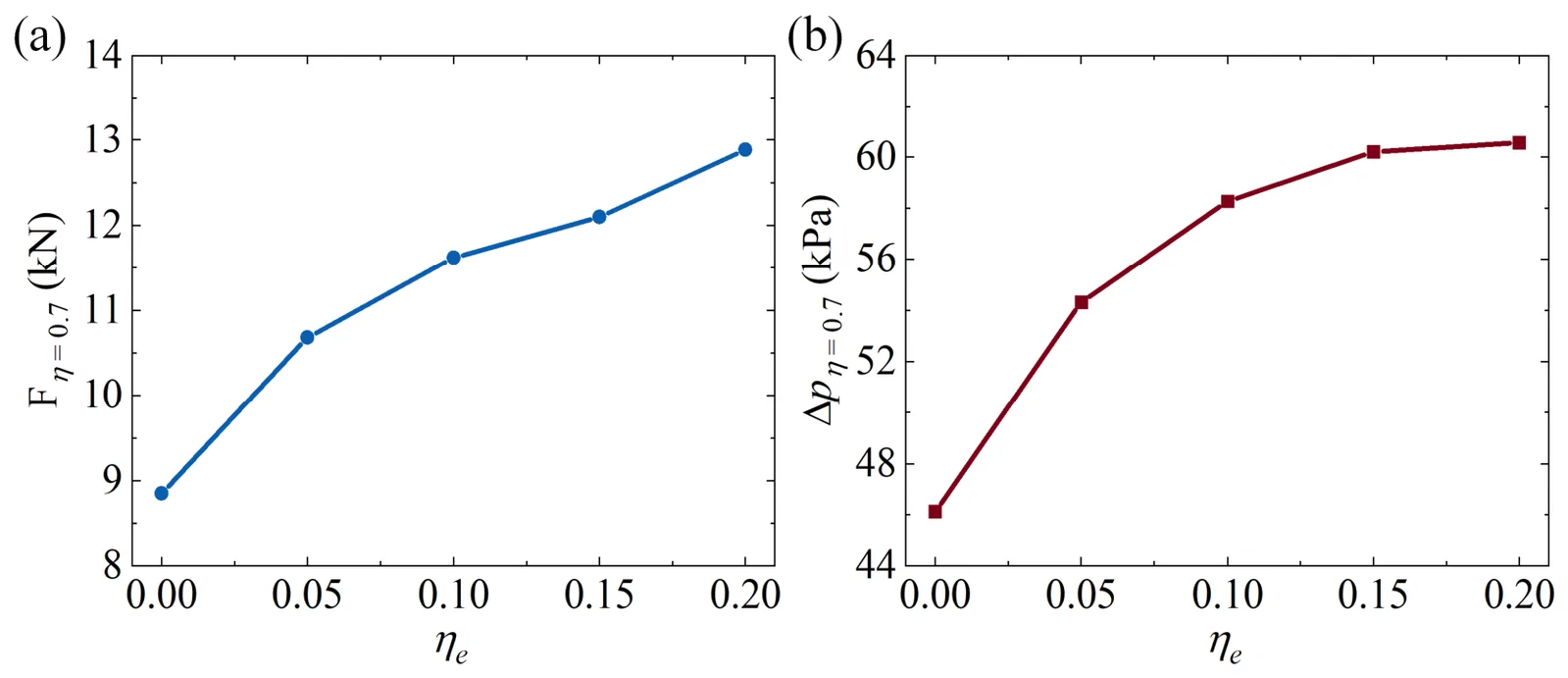
Effect of axial eccentricity on the terminal compression response at η=0.7: (**a**) rear-side force; (**b**) pressure increment.

The pressure increment also increases with $\eta_{e}$, but the growth progressively diminishes, as shown in Figure 17b. $\Delta p_{\eta =0.7}$ increases from 46.125 kPa at $\eta_{e}$ of 0 to 60.563 kPa at $\eta_{e}$ of 0.20, corresponding to an increase of 31.30%. In particular, increasing $\eta_{e}$ from 0.15 to 0.20 raises $\Delta p_{\eta =0.7}$ by only 0.61%, indicating that further movement of the loading platen toward the end of the airbag produces little additional cavity pressurization at the same compression ratio. Eccentric loading enhances the influence of asymmetric deformation and thereby increases both $F_{\eta =0.7}$ and $\Delta p_{\eta =0.7}$, with a greater effect when the loading position initially departs from the centre.

## 5. Conclusions

This study investigates the localized quasi-static compression response of a three-layer flexible anti-collision airbag comprising an inner TPU layer and two outer UHMWPE layers. Anisotropic hyperelastic models are developed and identified using uniaxial tensile tests. The finite element model is validated against the quasi-static compression tests and subsequently used to examine the effects of loading rate, axial loading length ratio, airbag aspect ratio, and axial eccentricity. The main conclusions are as follows:

(1) The tensile responses of TPU and UHMWPE vary with the material direction. TPU shows similar peak nominal stresses in the warp and weft directions but greater deformation capacity in the weft direction, whereas UHMWPE exhibits pronounced differences in deformation range and stiffness evolution between the two directions. The comparison of the six model combinations supports the selection of a Y3 isotropic basis with a weak orthotropic correction for TPU and an NH isotropic basis with a strong orthotropic correction for UHMWPE.
(2) The selected models reproduce the tensile response in both material directions and accurately predict the compression response of the airbag. The numerical force and pressure responses are in good agreement with the compression tests, with R^2^ > 0.985 and maximum relative differences of 4.183% and 0.348% at η of 0.7, respectively. These results confirm that the identified directional material behaviour and airbag model are applicable to the investigated deformation range.
(3) The compression tests show that increasing $p_{0}$ from 0.12 to 0.14 MPa raises the rear-side force and internal pressure at η=0.7 by approximately 51.1% and 19.7%, whereas increasing the loading rate from 5 to 50 mm/min changes the corresponding responses by only 2.81% and 0.58%, respectively. Numerical analyses further show that $F_{\eta =0.7}$ and $\Delta p_{\eta =0.7}$ remain nearly unchanged for prescribed loading rates up to 10 m/min. At higher rates, the force increases without a corresponding pressure rise, indicating an inertial departure from the numerical quasi-static response.
(4) The parametric analyses show that increasing the axial loading length ratio $\eta_{L}$ raises $F_{\eta =0.7}$ mainly by enlarging the loaded axial region, while $F_{\eta =0.7}/L_{P}$ remains nearly constant. Under the constant $\eta_{L}$ of 0.4, increasing the airbag aspect ratio Λ raises $F_{\eta =0.7}$, while $F_{\eta =0.7}/L_{P}$ and $\Delta p_{\eta =0.7}$ decrease. Increasing the axial eccentricity $\eta_{e}$ raises both $F_{\eta =0.7}$ and $\Delta p_{\eta =0.7}$, with the largest increments occurring when the loading position first departs from the centre.

However, this study focuses on dry conditions and compression ratios up to 0.7, and no visible material damage is observed. Since the material parameters are identified from uniaxial tests, independent predictive capability under biaxial tension and in-plane shear, particularly for the strongly orthotropic UHMWPE layer, remains to be established. Future research should consider the effects of the surrounding water and extend the constitutive framework to account for material damage and strain-rate-dependent behaviour. Furthermore, the local response of webbings under loading conditions involving large strain, pronounced nonlinearity, or seam damage also requires further investigation.

## Figures and Tables

**Table 1:** Identified parameters of the hyperelastic model for TPU.

Parameter	$C_{10}$	$C_{20}$	$C_{30}$	$k_{12}$	$k_{13}$
Value	44.02	247.79	70.60	−12.72	−85.77
Parameter	$k_{22}$	$k_{23}$	$k_{33}$	$k_{55}$	$k_{66}$
Value	−218.36	−88.72	−284.65	−34.47	−3.05

Note: All parameters are expressed in MPa. Values are rounded to two decimal places for presentation; the unrounded identified parameters are retained in the numerical calculations.

**Table 2:** Identified parameters of the hyperelastic model for UHMWPE.

Parameter	$C_{10}$	$A_{1}$		$B_{1}$		$D_{1}$
Value	20.49	53.69		1.40		1.74
Parameter	$A_{2}$		$B_{2}$		$D_{2}$	
Value	20.00		1.25		0.29	

Note: Parameters $A_{i}$ and $B_{i}$ are dimensionless, whereas $C_{10}$ and $D_{i}$ are expressed in MPa.

**Table 3:** Comparison of the experimental and finite element responses.

Initial Pressure (MPa)	Loading Rate (mm/min)	Test Resultat η=0.7		FE Resultat η=0.7	Difference (%)	R^2^
0.12	5	Force	5.921	6.135	3.610	0.9906
		Pressure	0.15630	0.15666	0.230	0.9995
0.13	5	Force	7.484	7.797	4.183	0.9854
		Pressure	0.16945	0.17004	0.348	0.9997
0.14	5	Force	8.948	9.223	3.078	0.9909
		Pressure	0.18344	0.18324	0.109	0.9996
0.12	50	Force	6.088	6.141	0.869	0.9932
		Pressure	0.15651	0.15655	0.026	0.9999

Note: All parameters of the force results at η=0.7 are expressed in kN, and the corresponding pressure results are expressed in MPa. The R^2^ values are calculated from the history curves over 0≤η≤0.7.

## Data Availability

The original contributions presented in this study are included in the article. Further inquiries can be directed to the corresponding author.
